# Differentiation‐associated urothelial cytochrome P450 oxidoreductase predicates the xenobiotic‐metabolizing activity of “luminal” muscle‐invasive bladder cancers

**DOI:** 10.1002/mc.22784

**Published:** 2018-02-01

**Authors:** Simon C. Baker, Volker M. Arlt, Radek Indra, Madeleine Joel, Marie Stiborová, Ian Eardley, Niaz Ahmad, Wolfgang Otto, Maximilian Burger, Peter Rubenwolf, David H. Phillips, Jennifer Southgate

**Affiliations:** ^1^ Jack Birch Unit of Molecular Carcinogenesis Department of Biology University of York Heslington York UK; ^2^ Department of Analytical, Environmental and Forensic Sciences MRC‐PHE Centre for Environment and Health King's College London Franklin‐Wilkins Building London UK; ^3^ NIHR Health Protection Research Unit in Health Impact of Environmental Hazards at King's College London in Partnership with Public Health England Franklin‐Wilkins Building London UK; ^4^ Faculty of Science Department of Biochemistry Charles University Albertov Prague Czech Republic; ^5^ St James's Hospital Leeds UK; ^6^ Department of Urology Regensburg University Medical Centre Regensburg Germany; ^7^ Department of Urology Frankfurt University Medical Center, Johann Wolfgang Goethe‐University Frankfurt am Main Germany

**Keywords:** aryl hydrocarbon receptor, CPR, CYP1A1, POR, urothelium

## Abstract

Extra‐hepatic metabolism of xenobiotics by epithelial tissues has evolved as a self‐defence mechanism but has potential to contribute to the local activation of carcinogens. Bladder epithelium (urothelium) is bathed in excreted urinary toxicants and pro‐carcinogens. This study reveals how differentiation affects cytochrome P450 (CYP) activity and the role of NADPH:P450 oxidoreductase (POR). CYP1A1 and CYP1B1 transcripts were inducible in normal human urothelial (NHU) cells maintained in both undifferentiated and functional barrier‐forming differentiated states in vitro. However, ethoxyresorufin O‐deethylation (EROD) activity, the generation of reactive BaP metabolites and BaP‐DNA adducts, were predominantly detected in differentiated NHU cell cultures. This gain‐of‐function was attributable to the expression of POR, an essential electron donor for all CYPs, which was significantly upregulated as part of urothelial differentiation. Immunohistology of muscle‐invasive bladder cancer (MIBC) revealed significant overall suppression of POR expression. Stratification of MIBC biopsies into “luminal” and “basal” groups, based on GATA3 and cytokeratin 5/6 labeling, showed POR over‐expression by a subgroup of the differentiated luminal tumors. In bladder cancer cell lines, CYP1‐activity was undetectable/low in basal POR^lo^ T24 and SCaBER cells and higher in the luminal POR over‐expressing RT4 and RT112 cells than in differentiated NHU cells, indicating that CYP‐function is related to differentiation status in bladder cancers. This study establishes POR as a predictive biomarker of metabolic potential. This has implications in bladder carcinogenesis for the hepatic versus local activation of carcinogens and as a functional predictor of the potential for MIBC to respond to prodrug therapies.

**Abbreviations**
ABS: adult bovine serum
AHR: aryl‐hydrocarbon receptor
BaP: benzo[a]pyrene
CYP: cytochrome P450
DMSO: dimethyl sulfoxide
EROD: ethoxyresorufin O‐deethylation
ITE: 2‐(1′H‐indole‐3′‐carbonyl)‐thiazole‐4‐carboxylic acid methyl ester
KSFMc: Keratinocyte Serum‐Free Medium complete
MIBC: muscle invasive bladder cancer
NHU: normal human urothelial (NHU) cells
PAH: polycyclic aromatic hydrocarbon
POR: NADPH:P450 oxidoreductase
RT‐qPCR: Reverse Transcribed‐quantitative Polymerase Chain Reaction
SNP: single nucleotide polymorphism
TMA: tissue micro‐array
TMS: 2,3′,4,5′‐tetramethoxystilbene

## 1 INTRODUCTION

The epithelial lining of the bladder and associated urinary tract, the urothelium, functions as a barrier to prevent reabsorption of the concentrated waste products of metabolism. The mechanisms used by the urothelium to survive a lifetime of toxin and toxicant exposure remain underexplored, although preliminary evidence of cytochrome P450 (CYP) transcript/activity supports a potentially important role in the urinary tract (reviewed in Ref. 1).

The aryl‐hydrocarbon receptor (AHR) is a transcription factor expressed constitutively by many epithelial tissues. In epidermal keratinocytes, AHR‐mediated transcription has been implicated in differentiation,^2^ although AHR is better known for regulating expression of a battery of *CYP* genes (including *CYP1A1*, *CYP1A2*, and *CYP1B1*) as part of Phase I metabolism in the detoxification of xenobiotics. CYP1‐mediated metabolism is particularly important for aromatic amines, which include the bladder pro‐carcinogens 2‐naphthylamine and 4‐aminobiphenyl, and polycyclic aromatic hydrocarbons (PAH), including benzo[*a*]pyrene (BaP; reviewed in Ref. 3). Phase I metabolism of xenobiotics by CYPs is often the first step in detoxification as it supports the Phase II conjugation reactions. However, Phase I metabolites are frequently reactive and studies have shown CYP1 enzyme function to be activating in the case of PAH pro‐carcinogens.^4^ *CYP1A1*, *CYP1B1*, and the AHR nuclear translocator (*ARNT*) have been identified as potential risk factors for human bladder cancer through association of single nucleotide polymorphisms (SNPs) with the disease,^5^, ^6^ but a robust case is lacking and it remains unclear whether the SNPs affect production of genotoxicants solely in the liver, or whether extra‐hepatic metabolism is involved.

Bladder cancer has a high prevalence of somatic mutations, a trait shared with cancers where chronic mutagen exposure plays a causal role (such as lung cancer and melanoma).^7^ Smoking is the main risk factor for bladder cancer with a hazard ratio of 2.33 for former smokers and 4.27 for current smokers.^8^ Following PAH exposure, incomplete hepatic metabolism leads to the excretion of bladder pro‐carcinogens in the urine^9^ where urothelial CYP‐activity could lead to DNA adduct formation and ultimately mutation. BaP is the major PAH in cigarette smoke and the bladder tissue of current smokers contains bulky DNA adducts.^10^

Acting in combination with microsomal epoxide hydrolase (EPHX1), the bioactivation of BaP to species capable of forming DNA adducts is performed most efficiently by CYP1A1 and CYP1B1.^11^ Studies of *Cyp1*‐knockout mice suggest Cyp1a1 is essential for detoxification of BaP in the epithelium of the gastrointestinal tract, but the bladder remains unstudied (reviewed in Ref. 12). CYP activity is driven by electron donation from the NADPH:CYP oxidoreductase (POR) and abundance of POR determines metabolic capacity in the CYP system.^13^, ^14^ POR is one member of the diflavin oxidoreductase family (nitric oxide synthase is the other) and is not specifically a reductase for the CYPs but can donate electrons to heme oxygenase among other enzymes (reviewed in Ref. 15). POR expression in normal and neoplastic urothelium of humans remains unquantified, although a SNP was recently associated with increased bladder cancer risk.^16^

Since the first report of CYP1 activity in rabbit bladders in 1985,^17^ there has only been fragmented study of AHR‐mediated metabolism in the urinary tract. Studies of non‐transformed cells have used either normal porcine urothelial cells (which demonstrate no ethoxyresorufin O‐deethylation (EROD), a measure for CYP1 enzyme activity)^18^ or human cells cultured from urinary sediments (which most likely originate from the kidney),^19^ limiting their relevance to human urothelium. It is therefore timely to establish whether CYP1 enzymes function in human urothelium and whether there is sufficient capacity to activate potential bladder carcinogens.

Normal (non‐neoplastic) human urothelium is not available in the quantities required to generate microsomes for the study of enzyme activity. The aim of this study was to establish the potential of bladder CYP1 metabolism; using in vitro cell culture models representing normal and neoplastic human urothelium in undifferentiated and differentiated states (reviewed in Ref. 20). Immunohistology was used to relate in vitro findings to the biology of human bladder tissue and muscle‐invasive bladder cancers (MIBC).

## 2 METHODS

### 2.1 Tissues and cells

All tissues were collected with appropriate Research Ethics Committee approvals and patient consents as required. In the UK, tissues were collected under the following NHS REC‐approved projects (Leeds East REC projects 99/095, 02/208, 00/22; York REC project 99/04/003, Newcastle & North Tyneside 1 REC 13/NE/0081). In Germany, tissue use was approved by the local research ethics committee of the University of Regensburg (IRB Number: 08/108) and materials (tissue sections) were transferred to York under a Materials Transfer Agreement.

Finite (non‐transformed) normal human urothelial (NHU) cell lines were established from surgical specimens (most commonly discarded ureteric tissues from renal cell carcinoma or transplant), as previously detailed.^21^ NHU cell lines were propagated as undifferentiated cultures in Keratinocyte Serum‐Free Medium containing recombinant epidermal growth factor and bovine pituitary extract (KSFMc; Invitrogen).^21^ For the studies described here, 15 independent cell lines were used between passages one and five. In the figures, each independent donor NHU cell line used has been given a unique symbol consistent between every panel (including supplementary).

Differentiation of in vitro‐propagated NHU cell cultures into barrier‐forming stratified urothelial cell sheets was performed as described in detail elsewhere.^22^ Briefly, NHU cells propagated in KSFMc were preconditioned for 5 days in 5% adult bovine serum (ABS) before harvesting and reseeding in same. After 24 h, the exogenous calcium concentration was increased to near‐physiological (2 mM) and cultures were maintained for 7 days before performing assays.

The endogenous natural ligand 2‐(1′H‐indole‐3′‐carbonyl)‐thiazole‐4‐carboxylic acid methyl ester (ITE; Tocris) was used to activate AHR in cell cultures at a concentration of 1 μM. NHU cell cultures were exposed to BaP (CAS no. 50‐32‐8; purity ≥96%; Sigma) at a concentration of 2 μM for 6 h. CYP1‐activity and BaP metabolism were inhibited by 2,3′,4,5′‐tetramethoxystilbene (TMS; Enzo Life Sciences) at 0.5–12 μM. Both compounds used DMSO as a vehicle and all controls contained a matched concentration of DMSO (not greater than 0.1% v/v).

For bladder cancer cell organ cultures,^23^ stromal tissue remaining after de‐epithelialization^21^ was dissected into 0.5 cm^2^ pieces and placed on Falcon membrane inserts (3 μm pore, Corning) for culture. RT4 (ECACC 91091914, sourced in 2000), RT112 (ECACC RT112/84 85061106, sourced in 2000) T24 (ATCC HTB‐4, sourced in 1999), and SCaBER (ATCC HTB‐3, sourced in 1999) cells were authenticated by short tandem repeat profiling using the PowerPlex16 System (Promega) in September 2016 and within five passages of use in this study (all cell lines were a perfect match to the ATCC genotype records). Bladder cancer cell lines were seeded onto the basement membrane of de‐epithelialized ureters and cultured at the air:liquid interface in DMEM:RPMI 1640 (50:50, v/v) with 5% fetal bovine serum for 4 weeks. Cancer cell organ cultures were fixed in formalin for 24 h, processed into paraffin wax and sectioned at 5 μm for immunoperoxidase labeling.

A tissue microarray (TMA) was constructed from formalin‐fixed paraffin wax‐embedded MIBC biopsies, exactly as described^24^ using tumor biopsies obtained from 61 non‐consecutive patients who underwent radical cystectomy for muscle‐invasive urothelial carcinoma of the urinary bladder in the Department of Urology, Regensburg University Medical Center, between 1998 and 2008. Median patient age was 71 (range: 55‐87) years. Eighty‐one percent of patients were male. Surgical specimens were assessed histopathologically by a single expert uropathologist for grading and staging based on the criteria of the 1973 WHO classification and 2010 TNM system.^25^, ^26^ Clinical characteristics of the patients are summarized in Supplementary Table S1. 1.5‐mm donor tissue cores were used, and the representativeness of the TMA was confirmed histopathologically by comparing the TMA and the original tissue sections for each tumor. Immunoperoxidase labeling of the MIBC TMAs was performed on 4‐μm sections mounted on poly‐L‐lysine‐coated glass slides and compared to a “normal” control group of bladders (non‐trigone cold‐cut biopsies) with no history of bladder malignancy. These tissues were from patients biopsied for a range of conditions including prostate cancer, stress incontinence, overactive bladder, and cystitis, some of which have been described previously.^27^

### 2.2 Indirect‐immunofluorescent labeling

To generate differentiated NHU cell cultures for immunolabeling, NHU cells preconditioned for 5 days in 5% ABS were passaged and seeded onto glass 12‐well slides at 3 × 10^4^ cells/well. After 24 h the calcium concentration was raised to 2 mM and the cultures maintained for 7 days, before treatment with ITE or a vehicle control. After 16 h, slides were fixed in methanol:acetone (1:1) for 30 s, air dried and rabbit anti‐AHR affinity‐purified polyclonal antibody was applied overnight at 4°C (1:500 dilution; Enzo Life Sciences, BML‐SA210). Unbound primary antibody was removed by washing in PBS and secondary antibody (Alexa‐594, Molecular Probes) was applied for 1 h at ambient temperature. Slides were washed in PBS, with 0.1 μg/mL Hoechst 33258 added to the penultimate wash, before mounting in antifade mountant and visualization by epifluorescence on a BX60 microscope (Olympus). Analysis was performed on images collected at a fixed exposure using TissueQuest Software (Tissue Gnostics).

### 2.3 Western blotting

Twenty micrograms of whole cell protein lysate was loaded into each gel track for electrotransfer to PVDF membranes with the full method provided in Supplementary Methods. The test antibodies used were anti‐AHR (1:2000, rabbit, Enzo Life Sciences, BML‐SA210), anti‐CYP1A1 (1:4000, rabbit, generous gift from Prof. F. Peter Guengerich, Vanderbilt University), anti‐POR (1:10 000, rabbit “CH60” a kind gift from Prof. Roland Wolf and Dr Colin Henderson, Dundee University).^28^ Homogeneous loading and transfer were evaluated using a β‐actin antibody (Sigma, Clone AC15, Mouse, 1:10 000 dilution). Detection of CYP1A1 and GAPDH protein was performed exactly as described.^29^ Densitometry was performed in all cases using Image Studio Lite Ver 5.0 software (LI‐COR). Cropped Western blots are shown in the main figures with full blots provided as Supplementary Figures S1 and S2.

### 2.4 Reverse transcribed‐quantitative polymerase chain reaction (RT‐qPCR)

Method and primers are provided in Supplementary Materials.

### 2.5 Ethoxyresorufin O‐deethylation (EROD) activity assays

EROD activity assays, used as a measure for CYP1 enzyme activity, were performed as described in detail elsewhere^30^ with minor modifications to convert the assay for black‐walled collagen‐coated glass coverslip‐bottomed 96‐well plates (BD Biosciences). Briefly, NHU/RT4/RT112/T24/SCaBER cells seeded at 6 × 10^4^/well were induced with ITE for predetermined times before washing in HEPES‐buffered saline (HBS; 138 mM NaCl, 5 mM KCl, 0.3 mM KH_2_PO_4_, 4 mM NaHCO_3_, 0.3 mM NaHPO_4_, 1 mM MgCl_2_, 2 mM CaCl_2_, and 10 mM HEPES pH 7.4) and treatment with 5 μM ethoxyresorufin (Cambridge Bioscience) in HBS. CYP1 enzyme activity was specifically inhibited by inclusion of TMS in the ethoxyresorufin/HBS. Plates were incubated for 75 min at 37°C; fluorescence was detected with 544 nm excitation and 590 nm emission filters using a POLARstar optima plate reader (BMG Labetch, housed in the Bioscience Technology Facility at York), and EROD activities were calculated using a (19‐625 nM) resorufin standard curve (*R* ^2^ = 0.996), which was corrected for cellular auto‐fluorescence. Following fluorescence measurement, plates were washed twice in phosphate‐buffered saline (PBS) and cells lysed in RIPA buffer (25 mM Hepes pH 7.4, 125 mM NaCl, 10 mM NaF, 10 mM sodium orthovanadate, 10 mM sodium pyrophosphate, 0.2% (w/v) SDS, 0.5% (w/v) sodium deoxycholate acid, 1% (w/v) Triton X–100, 1 mg/mL aprotinin, 10 mg/mL leupeptin, and 100 mg/mL phenylmethylsulphonyl fluoride) for a bicinchoninic acid (BCA) protein assay used for normalization (Fisher).

### 2.6 High performance liquid chromatography (HPLC) analysis of BaP metabolites

Culture medium from confluent 10 cm dishes of NHU cells was collected on ice, centrifuged at 4°C for 5 min at 300*g* and stored at −80°C until analysis. Per sample, 1 mL of medium was extracted twice with 1 mL of ethyl acetate. Extracts were evaporated and taken up in 30 μL methanol, of which 20 μL aliquots were injected on HPLC. HPLC analysis was performed using a HPLC Agilent 1100 System (Agilent Technologies) with a SunFireTM C18 reverse phase column (250 × 4.6 mm, 5 μm; Waters). The conditions used for the chromatographic separation of BaP metabolites were as follows: mobile phase (A) 50% acetonitrile in water (v/v), mobile phase and (B) 85% acetonitrile in water (v/v). The separation started with an isocratic elution of 1.4% of mobile phase B. Then a linear gradient to 98.5% of mobile phase B in 34.5 min was followed by isocratic elution for 6 min, a linear gradient from 98.5% to 1.4% of mobile phase B in 3 min, followed by an isocratic elution for 1.5 min. Total run time was 45 min at a flow rate of 1 mL/min. The metabolites were analyzed by fluorescence detection (excitation wavelength 381 nm, emission wavelength 431 nm). The two BaP metabolites analyzed, (±)‐*trans*‐7,8‐dihydroxy‐7,8‐dihydrobenzo[*a*]pyrene (BaP‐*t*‐7,8‐dihydrodiol) and (±)‐*r*‐7,*t*‐8,*t*‐9,*c*‐10‐tetrahydroxy‐7,8,9,10‐tetrahydrobenzo[*a*]pyrene (BaP‐tetrol‐I‐1), were identified using authentic standards which were synthesized as described.^29^

### 2.7 ^32^p‐postlabeling of BaP‐DNA adducts

Following collection of the medium, cultures were washed twice in cold D‐PBS (Gibco), scraped in 1 mL PBS, centrifuged at 800*g* for 5 min at 4°C and dry cell pellets were stored at −80°C until analysis. DNA was isolated from BaP‐treated cells using a standard phenol/chloroform extraction method. BaP‐DNA adduct formation was determined using the nuclease P1 digestion enrichment version of thin‐layer chromatography (TLC) and ^32^P‐postlabeling assay was carried out as described.^31^ Briefly, DNA samples (4 µg) were digested with micrococcal nuclease (288 mU; Sigma) and calf spleen phosphodiesterase (1.2 mU; MP Biomedical) and then enriched and labeled. Solvent conditions for the resolution of ^32^P‐labeled adducts on polyethyleneimine‐cellulose TLC were as described.^31^ Subsequently, TLC sheets were scanned using a Packard Instant Imager (Dowers Grove, IL) and DNA adducts (RAL, relative adduct labeling) were quantified from the adduct counts per minute (cpm), the specific activity of [γ‐^32^P]ATP (HP601PE; Hartmann Analytic) and the amount of DNA (pmol of DNA‐P) used. Results were expressed as DNA adducts per 10^8^ normal nucleotides. An external BPDE‐DNA standard^32^ was employed for identification of adducts in experimental samples.

### 2.8 Immunoperoxidase labeling

For immunolabeling of POR (1:4000, Mouse Clone F10, Santa Cruz, sc‐25270) and GATA3 (1:800, Rabbit antibody D13C9, Cell Signalling), heat‐mediated antigen retrieval was used (boiling for 10 min in 10 mM citric acid buffer [pH 6]); and labeling was performed using the Impress polymer detection kit according to the manufacturer's instructions (VectorLabs). All sections were counterstained with Mayer's haematoxylin, dehydrated and mounted in DPX (CellPath).

Cytokeratin 5/6 (KRT5/6; 1:50, M7237, Dako) labeling was performed on the Leica Bond 3 platform using Epitope Retrieval Solution 2 (AR9640; Leica Biosystems) for 30 minutes, a primary antibody application of 15 minutes, the Bond Polymer Refine Detection Kit (DS9800; Leica Biosystems) and Bond DAB enhancer (AR9432; Leica Biosystems) for 5 min.

Slides were imaged using an AxioScan.Z1 slide scanner (Zeiss). Labeling intensities were quantified using Histoquest software (v3.5, Tissue Gnostics) whereby regions containing >90% tumor cells were manually identified. For KRT5/6 quantification, the percentage area of tumor tissue labeled above the threshold intensity (of 50 arbitrary units) was calculated. An automated algorithm identified nuclei and cytoplasm based on the haematoxylin counterstain in order to perform the following analyses. Contaminating lymphocytes were removed from the analysis by gating out cells with a nuclear size smaller than 30 μm^2^. For GATA3 labeling, mean nuclear DAB intensity was quantified and a defined threshold (of labeling intensity 14; arbitrary units) was used to generate a labeling index of percentage positive nuclei within each tumor. For POR labeling, identified nuclei were used to support recognition of the surrounding cell body and cytoplasmic DAB intensity was quantified. The basal/luminal classification of the tumors based on GATA3 and KRT5/6 labeling was performed by reproducing the Logistic regression (LRA) and support vector machine (SVM) cut‐off lines derived previously by Dadhania et al.^33^

### 2.9 Statistical analysis

Data were assessed for statistical significance using InStat 3 or Prism 6 software (Graphpad). On all graphs statistical significance is represented as follows; *P < 0.05, **P < 0.01, and ***P < 0.001.

## 3 RESULTS

### 3.1 AHR in urothelial differentiation

**Figure 1:**
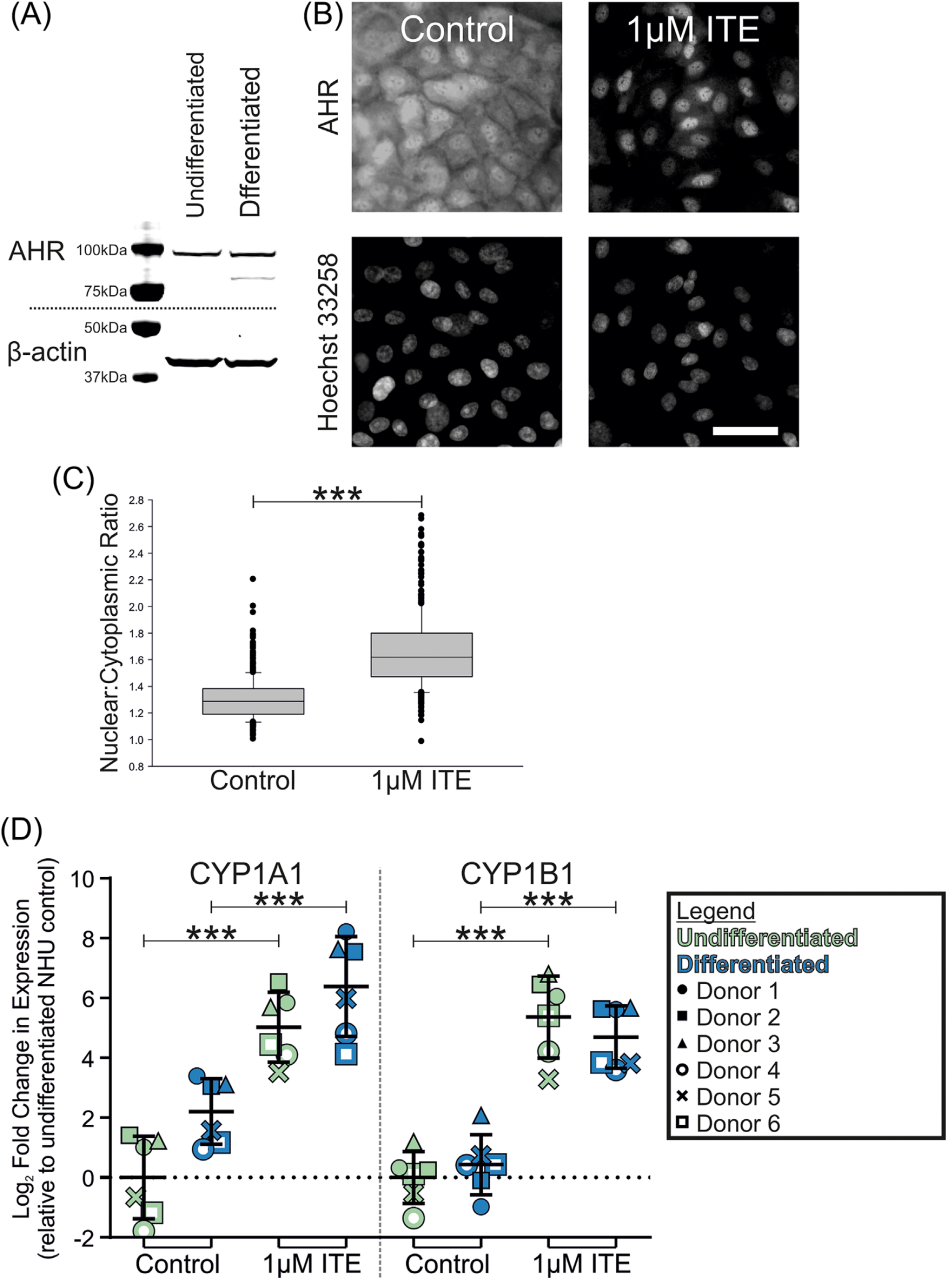
(A) Western blotting of whole cell lysates found AHR protein was detectable at similar abundance in undifferentiated and differentiated NHU cells. (B) Immunofluorescence labeling of differentiated NHU cells treated with either a vehicle control or 1 μM ITE for 16 h. AHR was distributed throughout cells in controls but was predominantly nuclear following ITE treatment. Scale bar = 100 μm. (C) Quantification of nuclear fluorescence intensity revealed a significant (*P *< 0.001, Student's *t*‐test) increase in nuclear:cytoplasmic ratio of AHR following 16 h exposure to 1 μM ITE. *n* = 3 experiments with >700 cells analyzed per treatment; boxes show median and 95% confidence interval with error bars showing SD and outliers shown as dots. (D) RT‐qPCR analysis of *CYP1A1* and *CYP1B1* transcript in NHU cell cultures treated with either vehicle control or 1 μM ITE for 24 h. In order to enable comparison of *CYP1* transcript abundance, RT‐qPCR data for urothelial cells are visualized in comparison with expression in whole normal human liver from pooled donors. ANOVA with Tukey‐Kramer post test showed ITE‐treatment provoked significant (*P* ≤ 0.001) increases in *CYP1A1* and *CYP1B1* gene expression. Results are presented as mean ± SD (*n* = 6 independent donor cell lines)

AHR protein was detected in similar abundance in both undifferentiated and differentiated NHU cell cultures (Figure 1A). AHR protein was observed to be widely distributed throughout the cell in differentiated NHU cells treated with a vehicle control; however, following exposure to 1 μM ITE for 16 h, AHR was significantly more abundant in the nucleus (Figures 1B and 1C).

AHR expression in epidermis has been attributed to a role in differentiation.^2^ *Ahr*‐knockout mice showed no perturbation of urothelial morphology or in the expression or distribution of proteins involved in urinary barrier function (including Claudin 5 and Uroplakin 3a, Supplementary Figure S3 and Supplementary Methods). Furthermore, trans‐epithelial electrical resistance monitored as a measure of barrier‐function in differentiated NHU cell cultures showed no significant change in response to ITE exposure throughout differentiation (mean resistance of 5007 vs 4978 Ω . cm^2^ for vehicle control and 1 μM ITE treatment, respectively; Student's *t*‐test *P* = 0.96, *n* = 6 cultures; Supplementary Figure S1 and Supplementary Methods).

### 3.2 Induction of CYP1A1 and CYP1B1 transcripts by AHR

The effect of the endogenous AHR ligand “ITE” on *CYP1* gene expression was studied in NHU cell lines from six independent donors. Significant (*P* ≤ 0.001) induction of *CYP1A1* and *CYP1B1* transcripts was observed in response to ITE in both undifferentiated (30 and 49‐fold, respectively) and differentiated (23 and 19‐fold, respectively) cultures compared to their respective vehicle controls (Figure 1D). Differentiated NHU cells showed preferential upregulation of *CYP1A1* transcript, while undifferentiated cells induced *CYP1B1* to a greater extent (Supplementary Figure S4). Differentiation of NHU cells itself induced a smaller increase in *CYP1A1* and *CYP1B1* transcript expression (4.1 and 1.5‐fold, respectively; Figure 1D). No expression of *CYP1A2* transcript was detected in NHU cells (data not shown).

### 3.3 Induction of EROD activity in NHU cells by AHR

**Figure 2:**
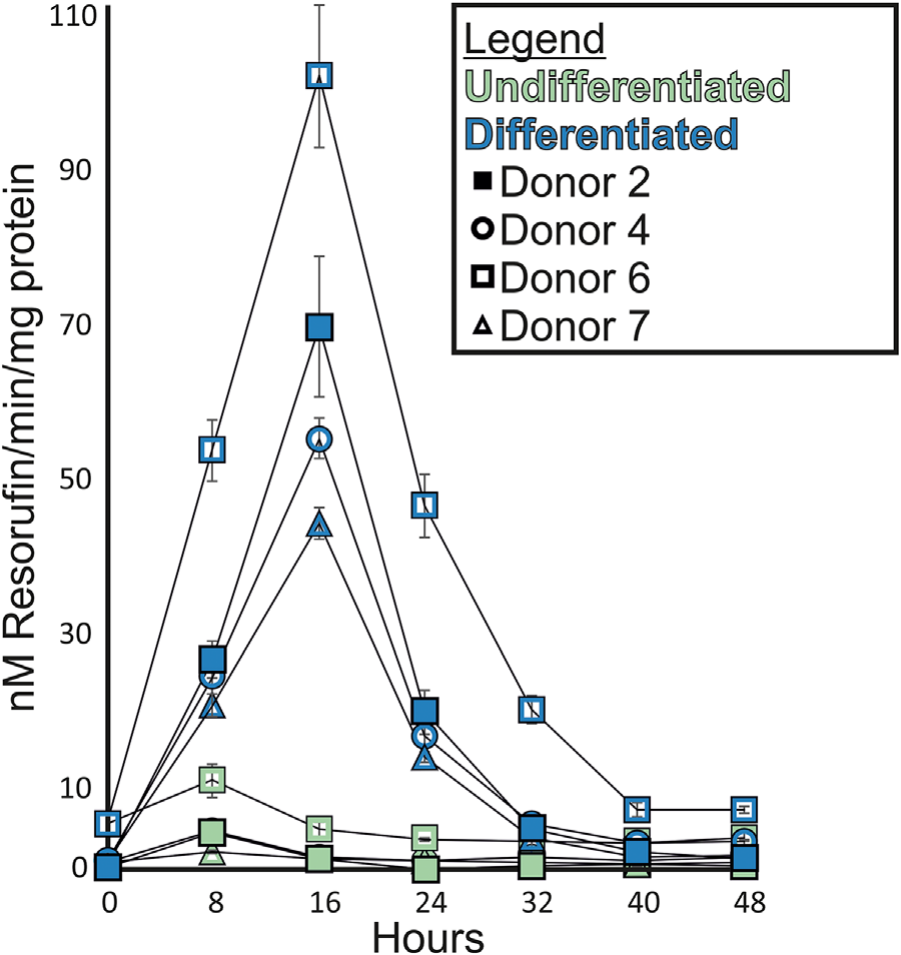
EROD activity assays demonstrated inducible CYP1‐enzyme function in differentiated NHU cells. EROD activity was not inducible in undifferentiated cultures. Differentiated NHU cells from four donors showed the same trend of 1 μM ITE‐induced EROD‐activity peaking at 16 h and returning to control levels by 40 h. Results are presented as mean ± SD (*n* = 6 replicates per independent donor cell line)

Basal EROD activity was barely detectable in NHU cells and was not inducible in undifferentiated NHU cells (Figure 2). EROD activity was rapidly induced in differentiated NHU cells by 1 μM ITE, with effects apparent by 8 h and a peak of activity at 16 h (mean 55‐fold increase, Figure 2). After 16 h (without replenishment of ITE), EROD activity in differentiated NHU cells began returning to baseline (Figure 2).

### 3.4 Inhibition of EROD activity by TMS

**Figure 3:**
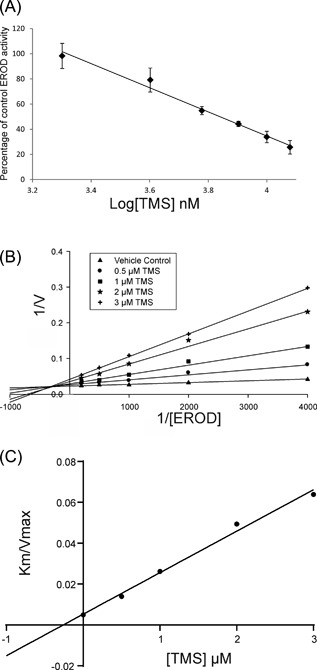
(A) Differentiated NHU cells treated for 16 h with 1 μM ITE were assessed for EROD activity in the presence of increasing concentrations of the specific CYP1 inhibitor TMS. Linear regression had an *R* ^2^ of 0.99 and the IC_50_ was 6.9 μM. Results are presented as mean ± SD (*n* = 3 independent donor cell lines with six replicates per donor). (B) Differentiated NHU cells treated for 16 h with 1 μM ITE were exposed to varying concentrations of ethoxyresorufin and TMS to generate a Lineweaver‐Burk plot. The experiment was repeated in three independent donor cell lines; this figure shows representative data from a single donor, values are the mean of duplicates. Trend‐lines were derived from Michaelis fitted data. (C) The Ki for TMS against differentiated NHU cell EROD activity was estimated as 0.26 μM by identifying the X‐axis intersect of the Km/Vmax trendline

EROD can be performed by all CYP1 family members with varying degrees of efficiency.^34^ As no *CYP1A2* transcript expression was observed in the urothelium, this enzyme was ruled out. Following a 16 h treatment with 1 μM ITE, induced CYP1 enzyme activity was inhibited by TMS in differentiated NHU cells from three donors with a mean IC_50_ of 6.9 μM (Figure 3A). To further characterize the inhibition of EROD‐activity by TMS, a Ki was derived from differentiated NHU cells following a 16 h treatment with 1 μM ITE. A Michaelis fit Lineweaver‐Burk plot demonstrated a mixed‐type inhibition where TMS had greater affinity for the free enzyme(s) than for the enzyme/substrate complex (Figure 3B). The mean estimated Ki for TMS against differentiated NHU cell CYP1 enzymes was 0.39 μM (±0.07) using quadratic analysis or 0.26 μM by identifying the X axis intersect of the Km/Vmax trendline (Figure 3C).

### 3.5 CYP1A1 and POR Western blotting before and after BaP exposure

**Figure 4:**
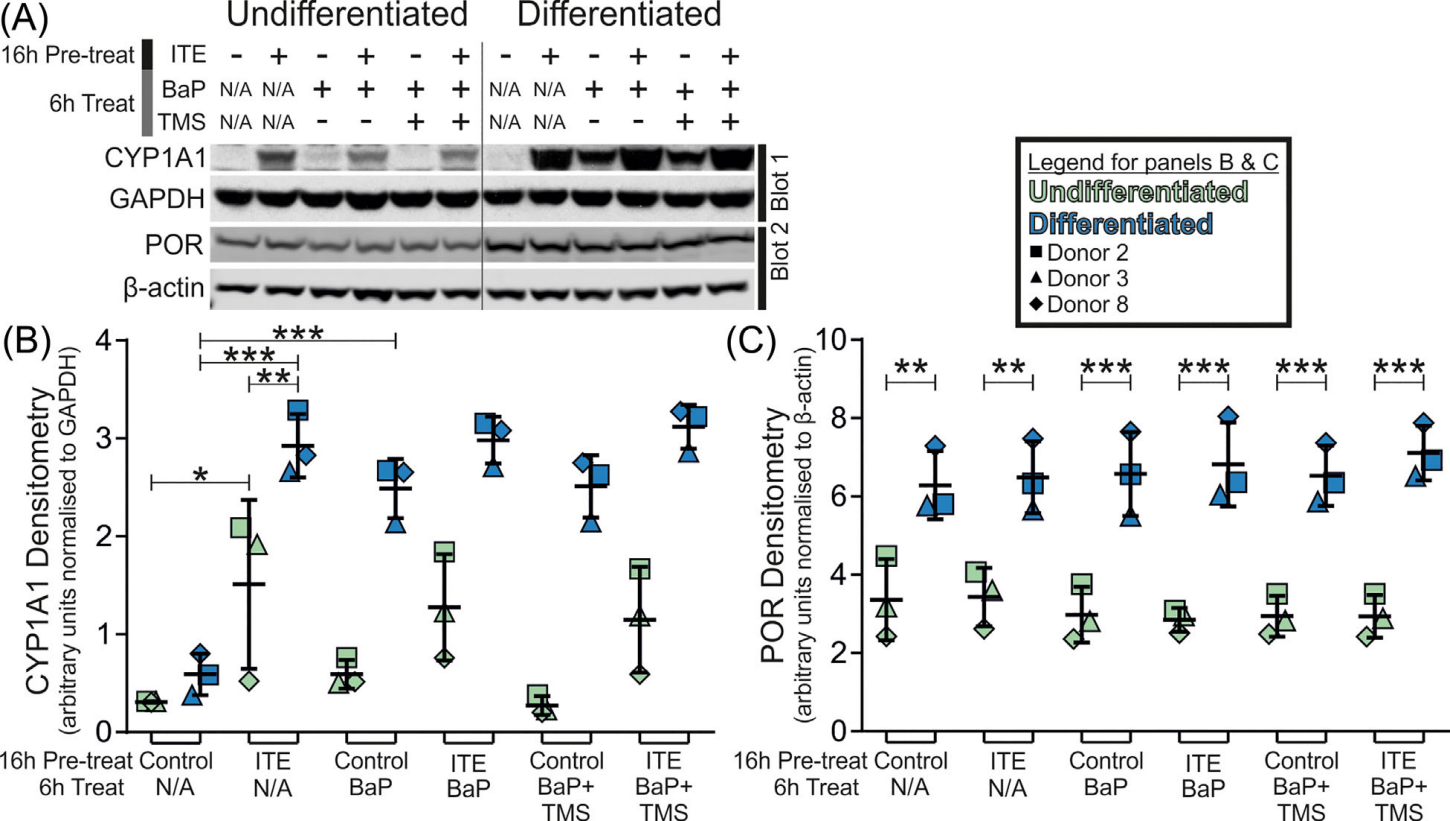
Western blotting of CYP1A1 normalized to GAPDH; and POR normalized to β‐actin in NHU cell cultures. CYP1A1 was significantly inducible in undifferentiated cells and differentiated cultures (4.9 and 5.0‐fold; *P *< 0.05 and *P *< 0.001, respectively) but reached significantly greater ITE‐induced abundance in differentiated cells (1.9‐fold *P* < 0.01). BaP exposure alone did not induce CYP1A1 expression in undifferentiated cells but did significantly in differentiated cultures (4.2‐fold; *P *< 0.001). POR expression was on average 2.2‐fold (*P* < 0.01) higher in differentiated cultures, but did not respond significantly to either ITE or BaP exposure. Results are presented as mean ± SD (*n* = 3 independent donor cell lines) with significance in expression assessed by ANOVA with Tukey‐Kramer post‐test. Representative Western blots shown from single donor. N/A, not applicable

NHU cells from three independent donors were lysed for whole‐cell Western blots to determine CYP1A1 and POR expression. Induction of CYP1A1 protein by 16 h pre‐treatment ±1 μM ITE confirmed RT‐qPCR data suggesting expression was low for control cells and significantly induced by ITE in both undifferentiated and differentiated states, but to a significantly greater extent in differentiated NHU cell cultures (Figure 4).

RT‐qPCR showed BaP exposure did not induce *CYP1* transcripts in undifferentiated NHU cells but did significantly in differentiated cultures (Supplementary Figure S5A). This result was confirmed for CYP1A1 at the protein level, where BaP exposure did not induce CYP1A1 expression in undifferentiated NHU cells, but did significantly in the differentiated cultures (Figure 4). No specific antibody could be found to support CYP1B1 detection.

Western blotting for POR established that abundance was not affected by ITE, TMS, or BaP exposure; but was elevated by differentiation of NHU cells (mean 2.1‐fold; Figure 4). RT‐qPCR of *POR* transcript confirmed differentiation of NHU cells from three donors increased *POR* transcript expression on average by 2.9‐fold; however, 1 μM ITE treatment had no significant effect on *POR* mRNA expression (Supplementary Figure S5B).

### 3.6 BaP metabolism

**Figure 5:**
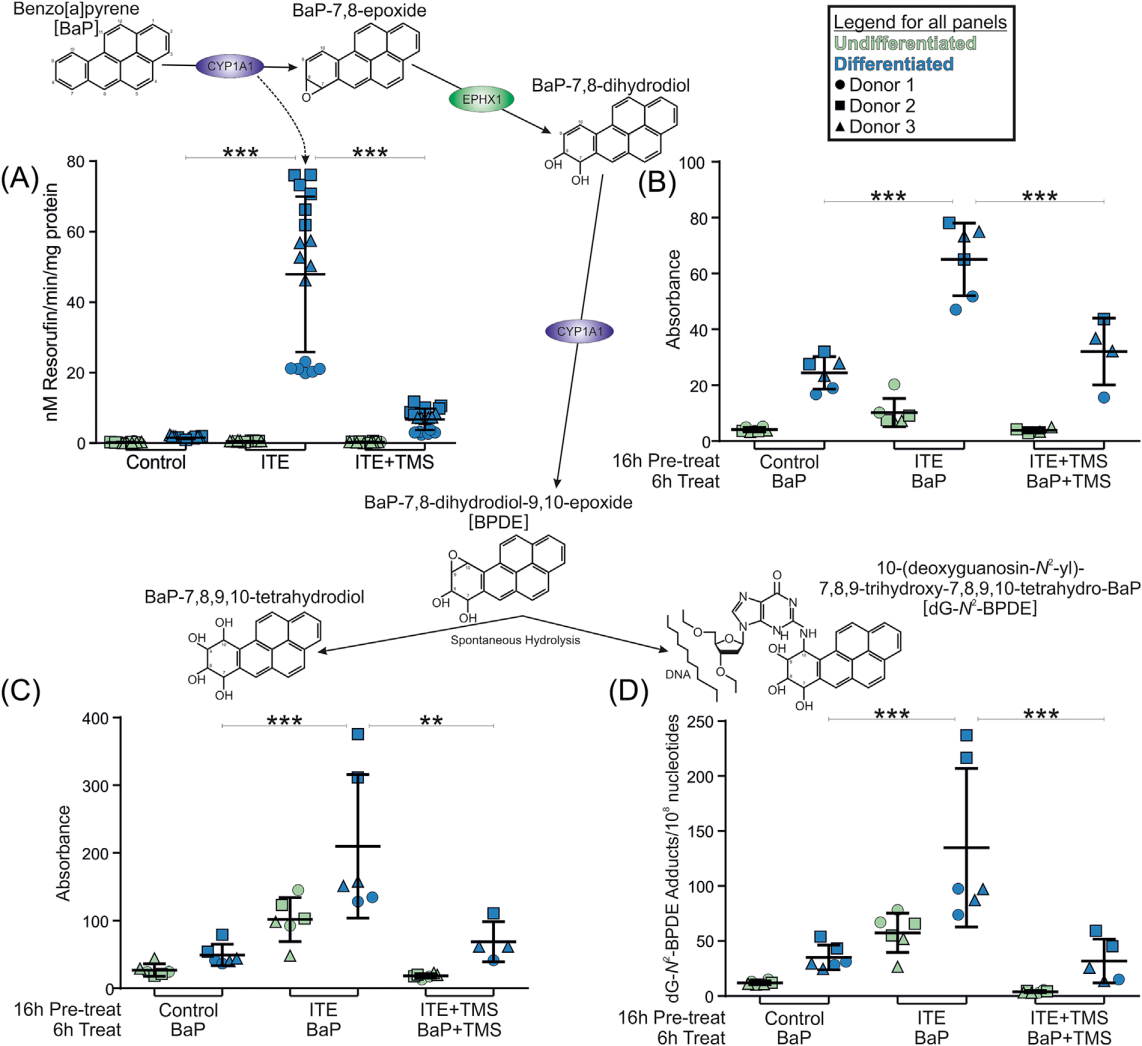
Schematic illustrating the metabolism of BaP by CYP1A1 with graphs showing data supporting active metabolism of BaP by differentiated NHU cells. (A) EROD activity was negligible in undifferentiated cultures. In differentiated NHU cells EROD was induced by 16 h 1 μM ITE pre‐treatment (42‐fold; *P *< 0.001) and inhibited to 14% of peak activity by 12 μM TMS (*P *< 0.0001). *n* = 3 independent donor cell lines; six replicate experiments per donor. (B) HPLC analysis of BaP‐7,8‐dihydrodiol in NHU cultures exposed to 2 μM BaP for 6 h. A total of 1 μM ITE pre‐treatment of differentiated NHU cells significantly increased the abundance of BaP‐7,8‐dihydrodiol in the culture medium compared to controls (2.7‐fold; *P *< 0.05) and 12 μM TMS reduced this to 46% (not significant). *n* = 3 independent donor cell lines; duplicate replicate experiments per donor. Representative chromatograms of the HPLC analysis are shown in Supplementary Figure 4. (C) HPLC analysis of BaP‐tetrol‐I‐1 in NHU cultures exposed to 2 μM BaP for 6 h. A total of 1 μM ITE pre‐treatment of differentiated NHU cells significantly increased the abundance of BaP‐tetrol in the culture medium compared to controls (4.1‐fold; *P *< 0.01) and 12 μM TMS significantly reduced this to 35% of induced levels (*P *< 0.05). Results are presented as mean ± SD (*n* = 3 independent donor cell lines; duplicate replicate experiments per donor). Representative chromatograms of the HPLC analysis are shown in Supplementary Figure 4. (D) ^32^P‐postlabeling analysis of BaP‐DNA adducts in NHU cultures exposed to 2 μM BaP for 6 h. A total of 1 μM ITE pre‐treatment of differentiated NHU cells significantly increased the number of dG‐*N* ^2^‐BPDE adducts per 10^8^ nucleotides compared to controls (3.7‐fold; *P* < 0.001) and 12 μM TMS significantly reduced this (to 21% of induced levels; *P* < 0.001). *n* = 3 independent donor cell lines; duplicate experiments per donor. Representative autoradiograms showing the DNA adduct profiles obtained by ^32^P‐postlabeling are shown in Supplementary Figure S5. For all panels, results are presented as mean ± SD, significance was assessed by ANOVA with Tukey‐Kramer post‐tests and cell lines from the same three donors were used for all graphs/biological end‐points

Cell lines established from three independent donors were monitored before (EROD, Figure 5A) and after BaP treatment (HPLC and ^32^P‐postlabeling, Figure 5B‐D). Monitoring EROD‐activity prior to BaP exposure confirmed significant induction of CYP1‐function in all three independent cell lines (mean 42‐fold) and showed that TMS provided significant inhibition of enzyme function in differentiated NHU cells (mean 14% of ITE‐induced activity, Figure 5A).

To establish whether urothelial CYP1 was capable of activating the known carcinogen BaP, cultures were exposed for 6 h and metabolites were measured in the culture medium by HPLC (Supplementary Figure S6). First, CYP1A1 oxidizes BaP to an epoxide (ie, BaP‐7,8‐epoxide), which is then converted to BaP‐7,8‐dihydrodiol by EPHX1. Further bioactivation by CYP1A1 leads to the reactive species, BaP‐7,8‐dihydrodiol‐9,10‐epoxide (BPDE) and BaP‐tetrol‐I‐1 is formed by spontaneous hydrolysis of BPDE. Peaks, referenced to standards, were observed and quantified for BaP‐7,8‐dihydrodiol (Figure 5B) and BaP‐tetrol‐I‐1 (Figure 5C). The formation of BaP metabolites mirrored the trends observed for EROD‐activity (Figure 5A); with metabolism greatest in differentiated NHU cell cultures, inducible by pre‐treatment with ITE (2.7 and 4.1‐fold for dihydrodiol and tetrol, respectively) and inhibited by TMS (to 46% and 35% for dihydrodiol and tetrol, respectively; Figures 5B and 5C).

The presence of BaP‐tetrol‐I‐1 in the medium of NHU cell cultures (Figure 5C) suggested that BPDE might also be forming the pre‐mutagenic DNA adducts (ie, 10‐(deoxyguanosin‐*N* ^2^‐yl)–7,8,9‐trihydroxy‐7,8,9,10‐tetrahydrobenzo[*a*]pyrene; “dG‐*N* ^2^‐BPDE” that have previously been observed in vitro and in vivo.^14^ Indeed the formation of dG‐*N* ^2^‐BPDE adducts was confirmed by ^32^P‐postlabeling (Supplementary Figure S7). No DNA adducts were detected in control (untreated) samples. Pre‐treatment of differentiated NHU cells with 1 μM ITE significantly increased BaP‐DNA adduct levels compared to controls (3.7‐fold) and 12 μM TMS significantly reduced this (to 21% of induced levels; Figure 5D). While the changes were not statistically significant, dG‐*N* ^2^‐BPDE adducts were formed in undifferentiated NHU cell cultures and adduct levels were both increased by ITE pre‐treatment and inhibited by TMS (Figure 5D). The correlation between EROD‐activity and BaP‐DNA adduct levels in the same three cell lines had an *R* ^2^ = 0.757 (Supplementary Figure S8).

### 3.7 Xenobiotic metabolism in bladder cancer

**Figure 6:**
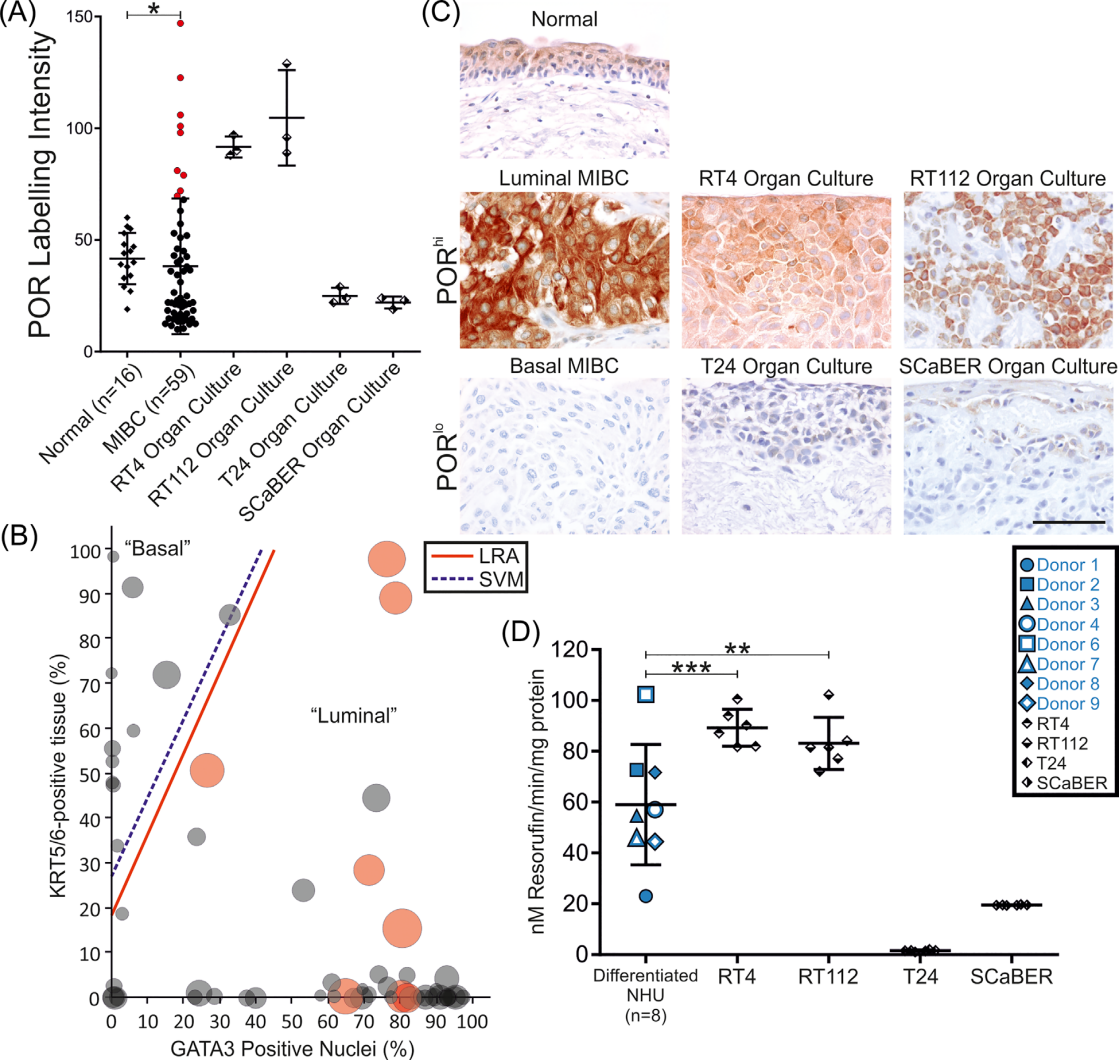
Analysis of NADPH:P450 oxidoreductase (POR) expression and CYP‐activity in bladder cancer. (A) Immunoperoxidase labeling of POR was quantified in a group of “normal” non‐neoplastic bladders (*n* = 16) and compared with muscle‐invasive bladder cancer (MIBC; *n* = 59) and organoid models of bladder cancer selected to represent POR suppressed (T24 and SCaBER) and over‐expressing (RT4 and RT112) tumors. MIBC showed significant (*P *< 0.05, Mann‐Whitney *U*‐test) suppression of mean POR expression; however, a group of outliers was noted which over‐expressed POR (open circles, representing 11.9% of cases). (B) Classification of MIBC as either luminal or basal based on quantification of GATA3 and KRT5/6 immunoperoxidase labeling was performed according to Dadhania et al^33^, reproducing the logistic regression analysis (LRA) and support vector machine (SVM) cut‐off lines derived in that study. All POR over‐expressing tumors (red circles) were classified as the more differentiated luminal type of MIBC. (C) Micrographs illustrating the POR immunoperoxidase labeling of normal bladder, MIBC, and cancer cell line (RT4, RT112, T24, and SCaBER) organ culture samples described in panel A. Scale bar represents 50 μm and applies to all images. (D) T24 and SCaBER cells generated peak EROD‐activity of 1.6 and 19.5 nM resorufin/min/mg, respectively following ITE exposure. By contrast, 1 μM ITE‐induced a peak EROD‐activity of 89.3 and 83.1 nM resorufin/min/mg in the POR over‐expressing RT4 and RT112 cell lines, respectively. RT4 and RT112 EROD was significantly higher than the mean of 60.0 nM resorufin/min/mg observed for differentiated NHU cells (*n* = 8 donors; each data point is the mean of six replicate cultures per donor)

Immunoperoxidase labeling of MIBC showed significant (*P *< 0.05) reduction in mean POR expression when compared to normal non‐neoplastic bladder urothelium (Figure 6 A). A sub‐group of MIBC (11.9% of tumors) was noted to over‐express POR, as compared to the normal bladders (open circles in Figure 6A). Both *CYP1A1* and *POR* transcript expression was significantly higher in luminal MIBC as compared with basal MIBC in The Cancer Genome Atlas cohort (Supplementary Figure S9). MIBCs were stratified into luminal and basal subgroups based on the GATA3 and cytokeratin 5/6 (KRT5/6) histology classifier first described by Dadhania et al^33^ that separates the two subtypes with 91% accuracy. The basal group had significantly less POR expression (*P *< 0.05, Mann‐Whitney Test) and the luminal group contained all the POR over‐expressing tumors identified previously (16.6% of luminal tumors; Figures 6B and 6C). The basal (Supplementary Figure S10) POR^lo^ bladder cancer cell lines T24 and SCaBER were used as in vitro models of POR suppression in MIBC (Figures 6A and 6C) and showed no/low inducible CYP1 enzyme activity, respectively (Figure 6D) despite *CYP1A1* and *CYP1B1* transcripts being ITE‐inducible (Supplementary Figure S11). As a model of the POR over‐expressing tumors (Figures 6A and 6C), the well‐differentiated/luminal (Supplementary Figure S10) RT4 and RT112 bladder cancer cell lines showed 1 μM ITE‐inducible EROD‐activity (Figure 6D). Mean peak EROD activity at 16 h was 60.0, 89.3, and 83.3 nM resorufin/min/mg for differentiated NHU, RT4 and RT112 cells, respectively (Figure 6D).

## 4 DISCUSSION

This study provides experimental and clinical evidence that CYP‐activity by normal urothelium is reliant on the differentiation‐dependent expression of POR, thereby defining the CYP‐capacity of different neoplastic programmes. POR abundance has been shown to influence CYP2B6‐mediated bioactivation of cyclophosphamide in patients^13^ and total CYP‐mediated metabolism in mice,^14^ and combined with this study suggests POR can be used as a biomarker of total CYP‐capacity in tissues. MIBC showed an overall suppression of POR; this was exemplified by the basal‐type T24 and SCaBER cell lines, which showed no EROD‐activity even though *CYP1* transcripts were inducible (Supplementary Figure S11), suggesting an overall loss of functional CYP‐activity in basal MIBC. By contrast, a subset of MIBC was identified that over‐expressed POR relative to normal bladder urothelium. In our series, this subgroup defined 16.6% of the luminal tumors. Using RT4 and RT112 cells as representative luminal POR over‐expressing bladder cancer cell lines, it was confirmed that these cells showed greater induced peak metabolic activity than achieved by differentiated NHU cells. These observations are important in associating functional, drug‐metabolizing activity to histologically‐defined tumor sub‐groups. Several CYP1‐activated therapies are in development to target epithelial tumors^35^, ^36^, ^37^ and POR is thought to be critical to the activation of some hypoxia‐activated prodrugs,^38^ offering potential for future trials targeting the POR^hi^ group of MIBC patients we report here.

The evidence presented here for CYP1A1 function in human urothelium and its role in BaP metabolism builds on earlier work showing BaP metabolism by organ cultures of human bladder tissue.^39^, ^40^ These earlier studies demonstrated that the tissue had capacity to metabolize BaP, but without confirming the cell type responsible due to the heterogeneous nature of the cell types present. In particular, it was noted that the bladder was the most active BaP metabolizer of all the human explant tissues tested at that point.^40^

Our study has shown *CYP1A1* and *CYP1B1* transcript expression by human urothelium and confirmed CYP1A1 protein, although no suitable antibody could be found for CYP1B1. Studies of purified enzymes show that CYP1A1 is more efficient at EROD (12‐fold) and BaP hydroxylation (2‐7‐fold) than CYP1B1.^34^, ^41^ Based on the greater induction of transcript in NHU cells and the greater efficiency of CYP1A1 to perform the reactions studied here, we believe that CYP1A1 is the critical enzyme in urothelial activation of BaP.

The potential for activation of pro‐carcinogens by human urothelium observed in this study provides a local mechanism for SNPs previously linked to bladder cancer (*CYP1A1*, *CYP1B1*, and *ARNT* 5, 6 and, more recently, *POR*).^16^ Despite strong evidence for the association of smoking and occupational BaP exposure with bladder cancer and the support in this study for urothelial activation of PAHs, the smoking‐associated mutational signature seen in lung cancer has not been observed in bladder cancer.^7^, ^42^ This may be due to the tissue‐specific nature of extra‐hepatic CYP activity (reviewed in Ref. 1) and the role of other CYPs that might metabolize BaP^11^ in human urothelium. This conclusion is supported by the efficacy of TMS inhibition which reduced EROD activity to 14%, BaP‐7,8‐dihydrodiol formation only to 46%, BaP‐tetrol formation to 35%, and BaP‐DNA adduct formation to 21% relative to ITE‐induced cells; suggesting that other enzymes not inhibited by TMS may play a role in BaP metabolism by the urothelium.

Natural AHR ligands, such as the tryptophan metabolites indigo and indirubin (which are structurally similar to ITE), have been detected in urine from healthy patients^43^ making them potential drivers of urothelial CYP1 activity. It is therefore theoretically possible that the coincidence of endogenous AHR ligands with urinary pro‐carcinogens in the bladder might contribute to accelerated carcinogenesis in some patients

No evidence was found to support a role for AHR in urothelial cytodifferentiation, which is known to be primarily driven by peroxisome proliferator‐activated receptor γ (PPARγ).^44^ By contrast, adipocyte differentiation is also mediated by PPARγ and during that process AHR expression is lost^45^ suggesting a functional maintenance of AHR by differentiated urothelium.

To what extent urothelial (rather than hepatic) metabolism generates the mutagens that drive cancer initiation remains to be established in future studies. However, the temptation to resort to in vivo studies is flawed by the poor homology between human CYP1A1 protein and the rat/mouse/pig orthologs in the BaP interacting region (80.6%, 80.9%, and 82.5%, respectively; Supplementary Figure S12). In particular, Asn‐222 of *CYP1A1* has evolved as a negatively charged aspartic acid in rats, mice, and pigs. Asn‐222 lies centrally in the five‐residue disruption of helix F that is unique to CYP1A1 and appears to modulate substrate movement, binding, and orientation.^46^ In addition, Asn‐222 is thought to be within 5Å of BaP when bound by CYP1A1 and involved in an extensive hydrogen bonding network that stabilizes the binding pocket.^46^ Taken together with the differences in rodent urinary tract physiology, with short duration of urinary storage, differences in urothelial cell cycle regulation and a low threshold for cancer initiation,^47^ this limits the validity of extrapolating rodent in vivo studies to humans. In this study, a more direct human line of evidence was pursued from 2D cultures of normal and malignant urothelium, via organoids to primary MIBC, offering an alternative route to the in vitro‐in vivo paradigm.

## 5 CONCLUSION

It has been thought for decades that the metabolism of pro‐carcinogens occurred in the liver and that bladder cancer was caused by the hydrolysis of conjugated metabolites in the urine or by enzymatic deconjugation in the urothelium. This study demonstrates the capacity of functionally‐differentiated normal human urothelium to activate the pro‐carcinogen BaP locally to active intermediates capable of forming DNA adducts (ie, dG‐*N* ^2^‐BPDE). The relative contributions of hepatic and urothelial metabolism to carcinogen activation should be re‐evaluated to better understand their relative roles in bladder cancer initiation. Furthermore, the association between differentiation and xenobiotic metabolism is maintained in a sub‐group of POR‐overexpressing luminal MIBC of predicted high metabolic potential, suggesting these patients as candidates for prodrug therapies.

## AUTHORS' CONTRIBUTION

SB, VA, PR, DP, and JS were involved in the conception and design of the study. SB, VA, RI, WO, and MJ acquired the data. All authors were involved in drafting and approving the final manuscript.

## Supporting information

Additional Supporting Information may be found online in the supporting information tab for this article.

**Figure S1**. Full western blots for Figure 4A&B.
**Figure S2**. Full western blots for Figure 4A&C.
**Figure S3**. Representative images of immunoperoxidase labelling for AHR and the urothelial barrier proteins, claudin 5 and uroplakin 3a.
**Figure S4**. Differential regulation of *CYP1A1* and *CYP1B1* transcripts by AHR stimulation of NHU cells in different states.
**Figure S5**. (A) RT‐qPCR demonstrated that 6 h 2 μM BaP exposure did not induce CYP1A1 or CYP1B1 transcripts in undifferentiated NHU cells but did significantly in differentiated cultures (p<0.01 and p<0.05 for CYP1A1 and CYP1B1, respectively; ANOVA with Tukey‐Kramer post‐tests).
**Figure S6**. Representative HPLC chromatograms of the BaP metabolites observed in medium samples exposed to NHU cells.
**Figure S7**. Autoradiographic profiles of DNA adducts, measured by ^32^P‐postlabelling, in undifferentiated and differentiated of NHU cells treated with BaP.
**Figure S8**. Linear regression analysis of EROD activity (Figure 5A) and dG‐N2‐BPDE adducts (Figure 5D) showing an R2 of 0.757.
**Figure S9**. CYP1A1 and POR transcript expression quantified from RNA sequencing data of the The Cancer Genome Atlas consortium and separated into basal and luminal subtypes based on the gene classifier reported by Choi et al. (55).
**Figure S10**. Heatmap of “UBC‐40” bladder cancer cell line gene array data (54) for the relative expression of Choi et al. (55) gene classifiers by RT4, RT112, T24 and SCaBER cells.
**Figure S11**. RT‐qPCR demonstrated that 24 h 1 μM ITE exposure induced CYP1A1 and CYP1B1 transcript in both RT4 and T24 cells.
**Figure S12**. Clustal Omega alignment of Ensembl protein sequences for the BaP interacting region of CYP1A1 in human (amino acids 115‐496 [49]; ENST00000379727.7), CL57BL6 mouse (ENSMUST00000216433.1), rat (ENSRNOT00000026473.4) and pig (ENSSSCT00000002135.3).

**Table S1**. Clinical characteristics and histological assessment of MIBC specimens included in the study.
