# Supplementary material for: Differentiation‐associated urothelial cytochrome P450 oxidoreductase predicates the xenobiotic‐metabolizing activity of “luminal” muscle‐invasive bladder cancers
Source: Mol Carcinog. 2018 Feb 1;57(5):606–18. doi: 10.1002/mc.22784 (PMC5900743; doi:10.1002/mc.22784)
Supplement: Supplementary file 1 — Figure S1. Full western blots for Figure 4A&B. Figure S2. Full western blots for Figure 4A&C. Figure S3. Representative images of immunoperoxidase labelling for AHR and the urothelial barrier proteins, claudin 5 and uroplakin 3a. Figure S4. Differential regulation of CYP1A1 and CYP1B1 transcripts by AHR stimulation of NHU cells in different states. Figure S5. (A) RT‐qPCR demonstrated that 6 h 2 μM BaP exposure did not induce CYP1A1 or CYP1B1 transcripts in undifferentiated NHU cells but did significantly in differentiated cultures (p<0.01 and p<0.05 for CYP1A1 and CYP1B1, respectively; ANOVA with Tukey‐Kramer post‐tests). Figure S6. Representative HPLC chromatograms of the BaP metabolites observed in medium samples exposed to NHU cells. Figure S7. Autoradiographic profiles of DNA adducts, measured by 32P‐postlabelling, in undifferentiated and differentiated of NHU cells treated with BaP. Figure S8. Linear regression analysis of EROD activity (Figure 5A) and dG‐N2‐BPDE adducts (Figure 5D) showing an R2 of 0.757. Figure S9. CYP1A1 and POR transcript expression quantified from RNA sequencing data of the The Cancer Genome Atlas consortium and separated into basal and luminal subtypes based on the gene classifier reported by Choi et al. (55). Figure S10. Heatmap of “UBC‐40” bladder cancer cell line gene array data (54) for the relative expression of Choi et al. (55) gene classifiers by RT4, RT112, T24 and SCaBER cells. Figure S11. RT‐qPCR demonstrated that 24 h 1 μM ITE exposure induced CYP1A1 and CYP1B1 transcript in both RT4 and T24 cells. Figure S12. Clustal Omega alignment of Ensembl protein sequences for the BaP interacting region of CYP1A1 in human (amino acids 115‐496 [49]; ENST00000379727.7), CL57BL6 mouse (ENSMUST00000216433.1), rat (ENSRNOT00000026473.4) and pig (ENSSSCT00000002135.3). [file MC-57-606-s001.pdf]

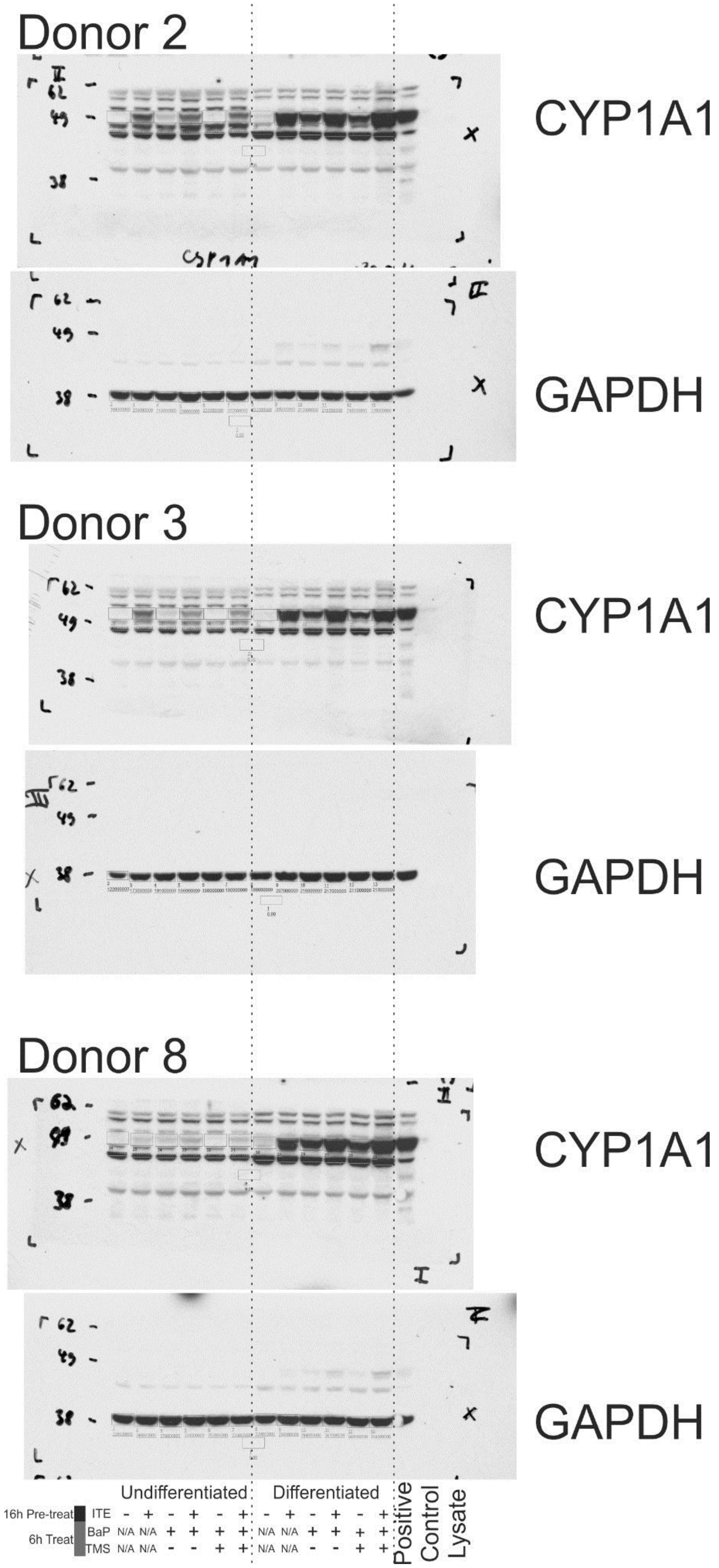

Supplementary Figure 1 – Full western blots for Figure 4A&B.

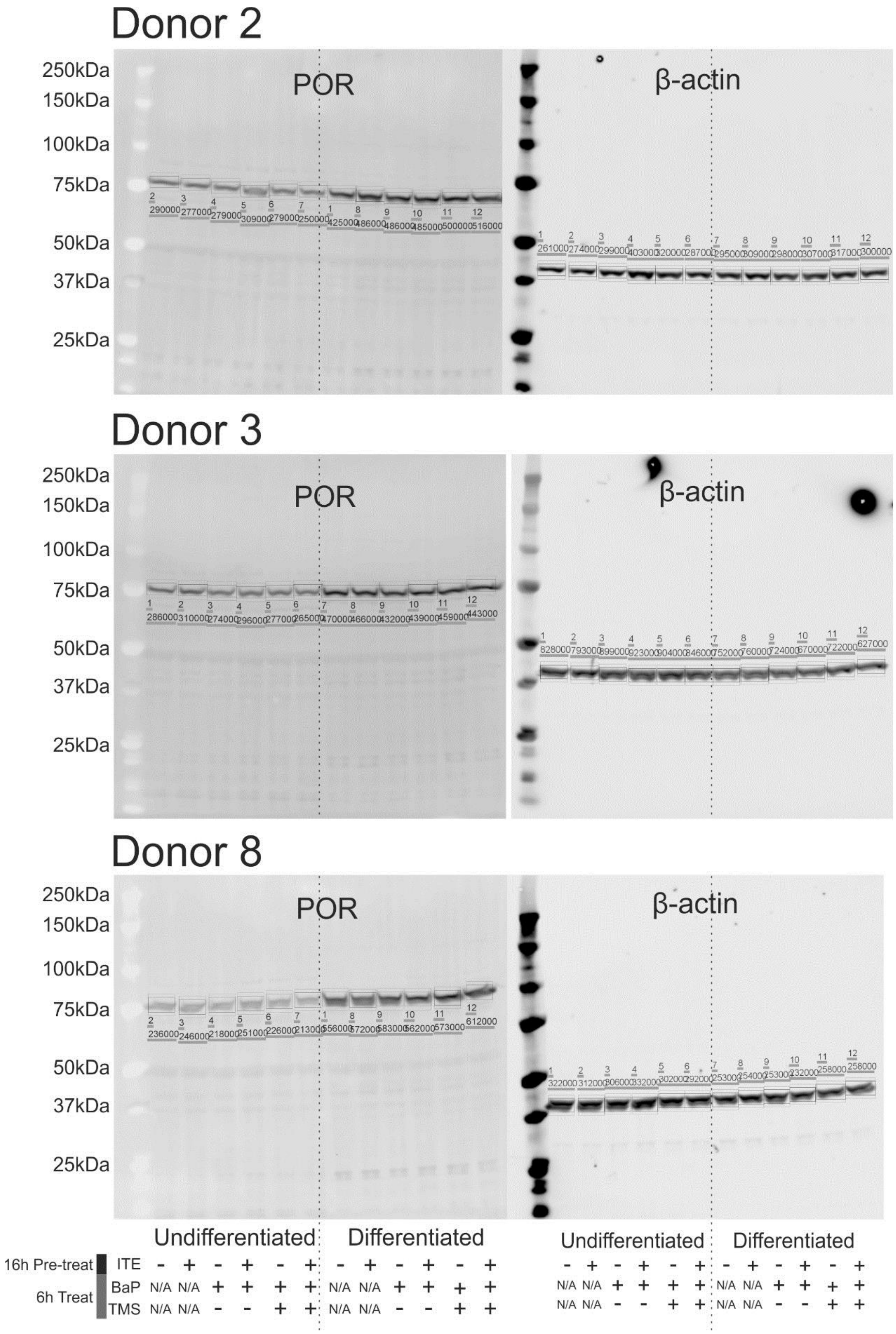

Supplementary Figure 2 – Full western blots for Figure 4A&C.

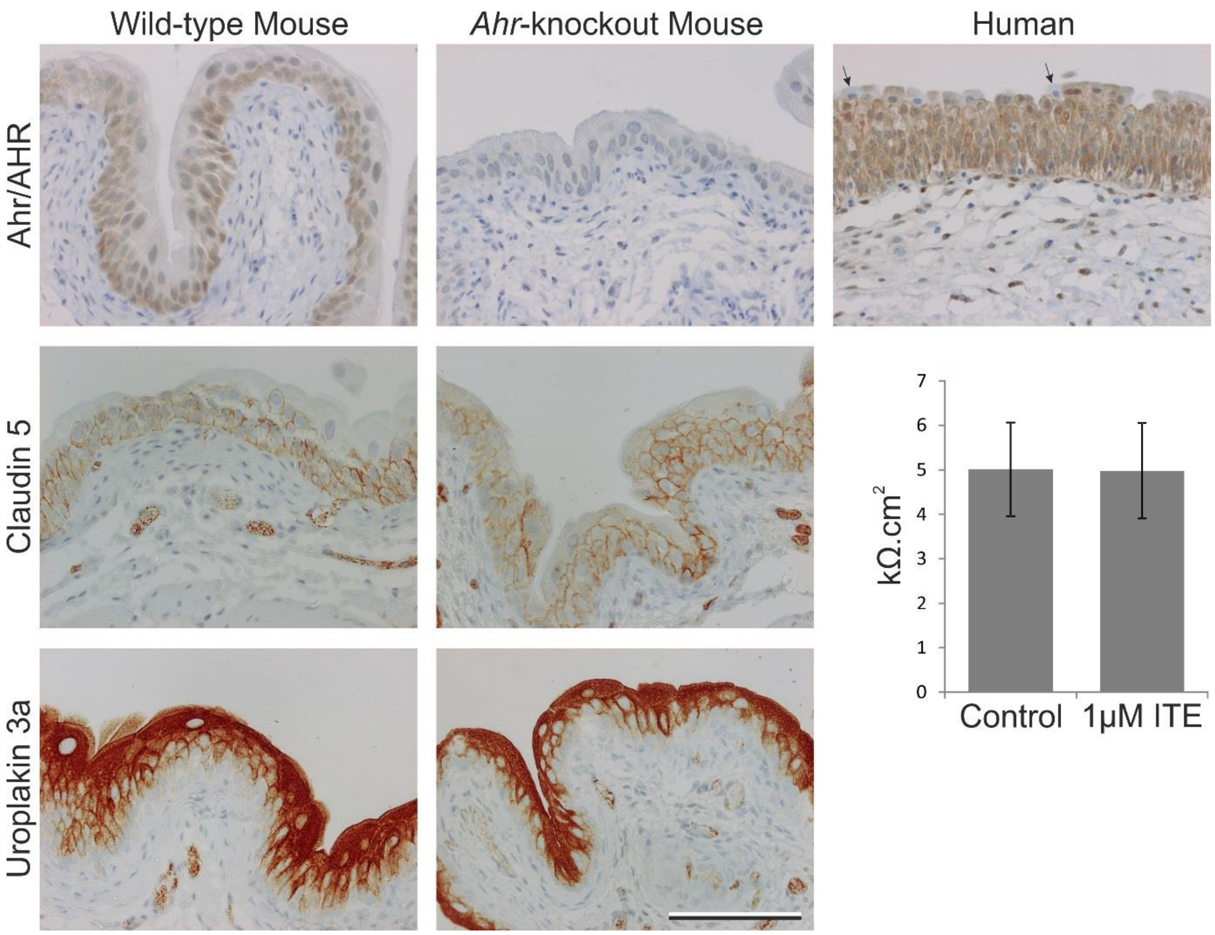

Supplementary Figure 3 - Representative images of immunoperoxidase labelling for AHR and the urothelial barrier proteins, claudin 5 and uroplakin 3a. AHR expression was confined to basal and intermediate cells of the wild-type mice and absent from the knockout. In human samples AHR expression was additionally observed in the superficial layer although occasional cells were negative (indicated with arrows). The knockout of AHR in mice resulted in no changes to either intensity or distribution of expression of urothelial differentiation markers. n = ≥6 animals, Scale bar = 100 µm. Graph shows TEER data for NHU cells differentiated in the presence of 1 M ITE, showing exposure to the AHR ligand had no significant (*p*=0.96, Student's *t*-test) effect on urothelial barrier function. Results are presented as mean ± SD (*n* = 6 experiments on a single normal cell line).

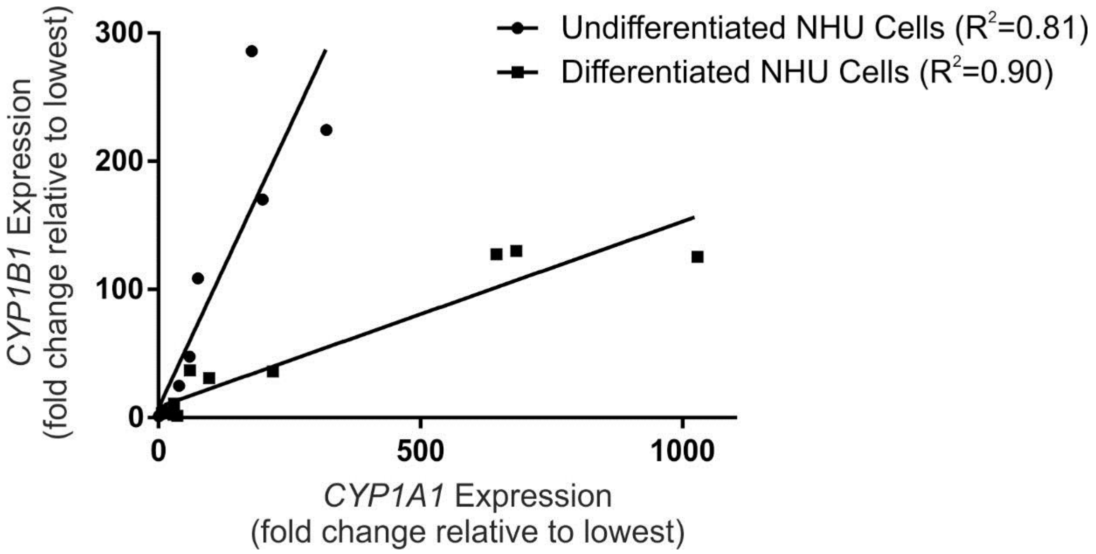

Supplementary Figure 4 – Differential regulation of *CYP1A1* and *CYP1B1* transcripts by AHR stimulation of NHU cells in different states. Undifferentiated and differentiated NHU cells exhibit a significant (p<0.0001) difference in preferentially upregulating either *CYP1B1* or *CYP1A1*, respectively. n = 6 donor cell lines in untreated and 1 µM ITE exposed (24 h) states.

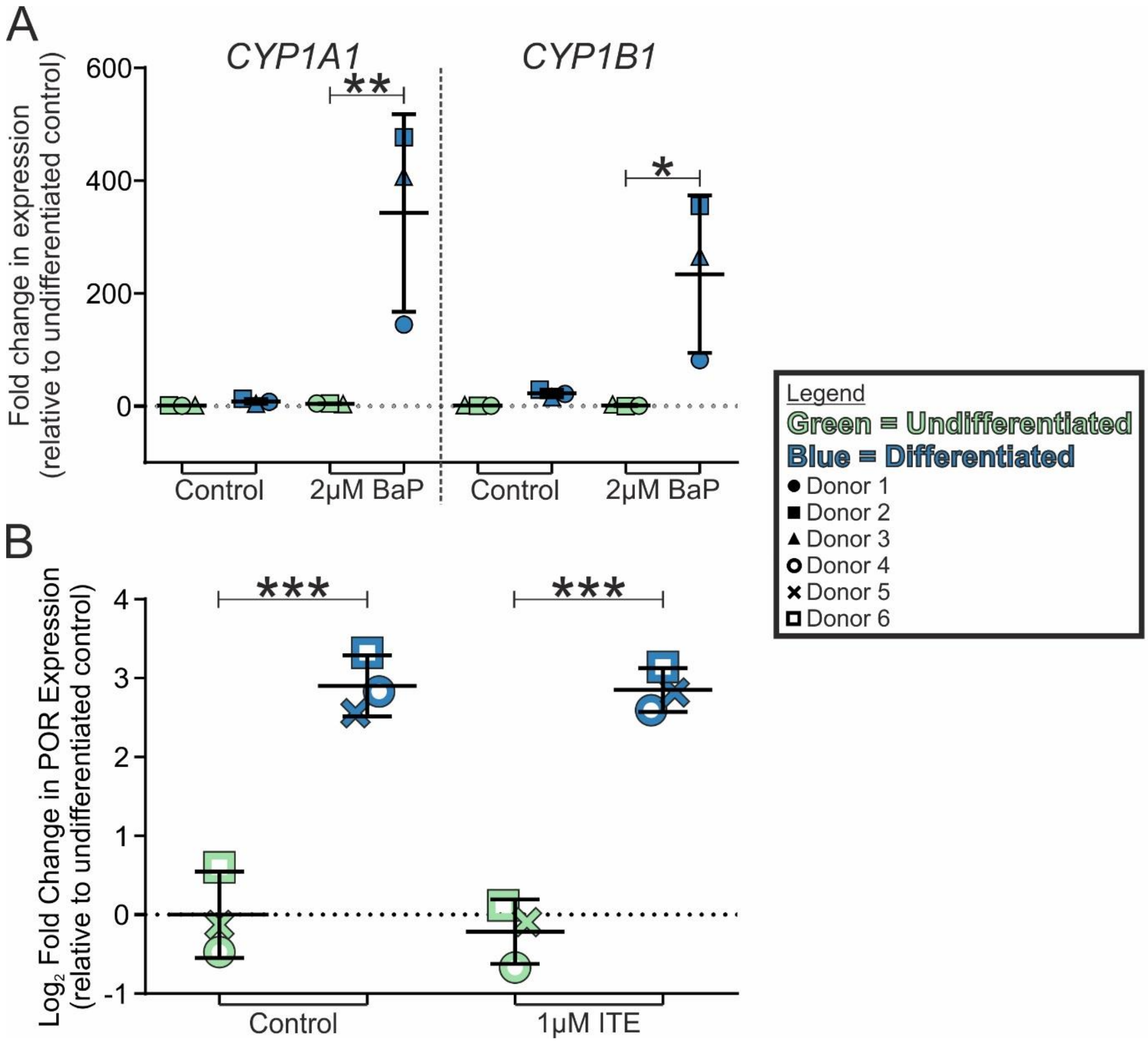

Supplementary Figure 5 – (A) RT-qPCR demonstrated that 6 h 2 µM BaP exposure did not induce *CYP1A1* or *CYP1B1* transcripts in undifferentiated NHU cells but did significantly in differentiated cultures (p<0.01 and p<0.05 for *CYP1A1* and *CYP1B1*, respectively; ANOVA with Tukey-Kramer post-tests). n = 3 independent donor cell lines, normalised to mean expression in the undifferentiated vehicle control cells for each gene and error bars = standard deviation.

(B) RT-qPCR of *POR* in undifferentiated and functionally-differentiated NHU cells treated with a vehicle control or 1 µM ITE for 24 h, normalised to mean expression in the undifferentiated vehicle control cells. No significant changes were observed in the expression of *POR* transcripts in response to ITE treatment. Data are expressed as Log_2 fold change, n = 3 independent donor cell lines, and error bars = standard deviation.

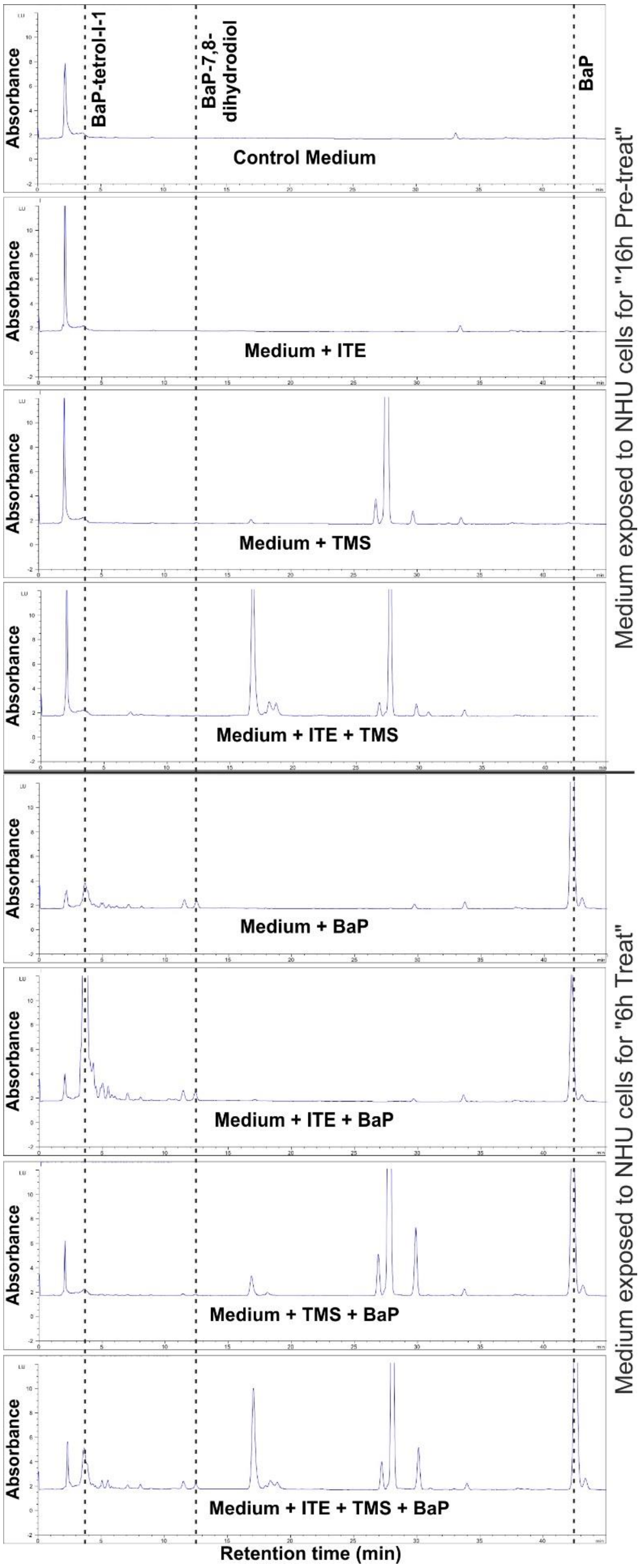

Supplementary Figure 6 - Representative HPLC chromatograms of the BaP metabolites observed in medium samples exposed to NHU cells.

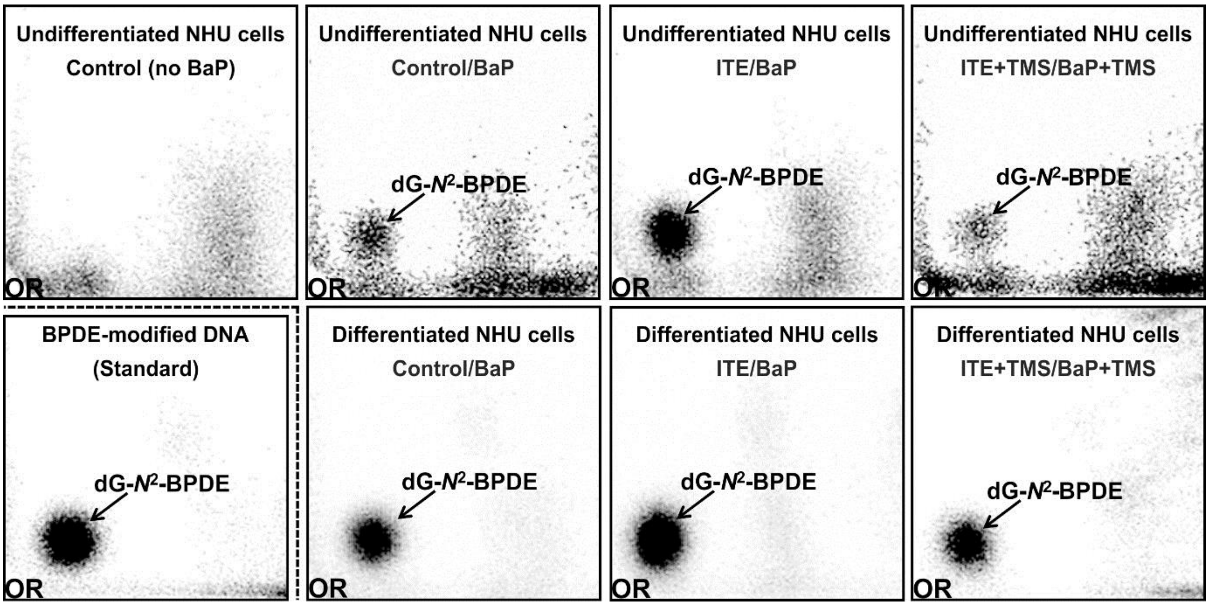

Supplementary Figure 7 – Autoradiographic profiles of DNA adducts, measured by ^{32}P-postlabelling, in undifferentiated and differentiated of NHU cells treated with BaP. Solvent conditions for the separation of BaP-derived DNA adducts were as follows: D1, 1.0 M sodium phosphate, pH 6.0; D3, 3.5 M lithium formate, 8.5 M urea, pH 3.5; D4, 0.8 M lithium chloride, 0.5 M Tris, 8.5 M urea, pH 8.0. The origins (OR), at the bottom left-hand corners, were cut off before exposure. The arrow indicates the 10-(deoxyguanosin-N^2-yl)-7,8,9-trihydroxy-7,8,9,10-tetrahydro-BaP (dG-N^2-BPDE).

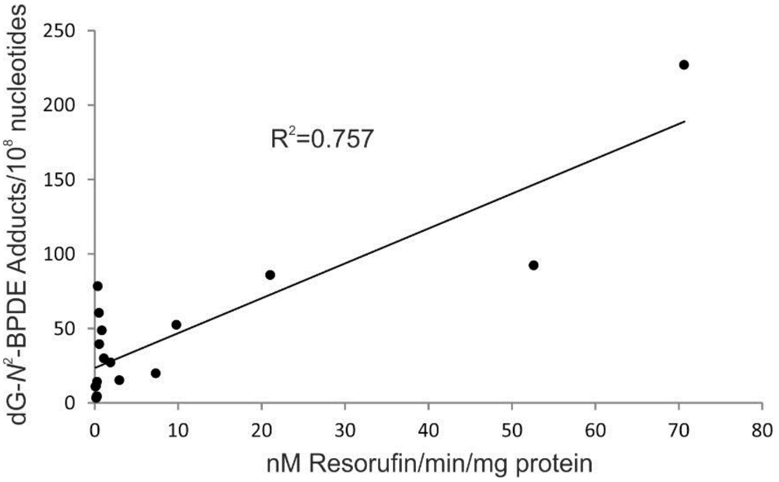

Supplementary Figure 8 – Linear regression analysis of EROD activity (Figure 5A) and dG-N^2-BPDE adducts (Figure 5D) showing an R^2 of 0.757. n = 16 experiments on cells from 3 independent donor cell lines in undifferentiated and differentiated states, with and without 16 h 1 µM ITE induction.

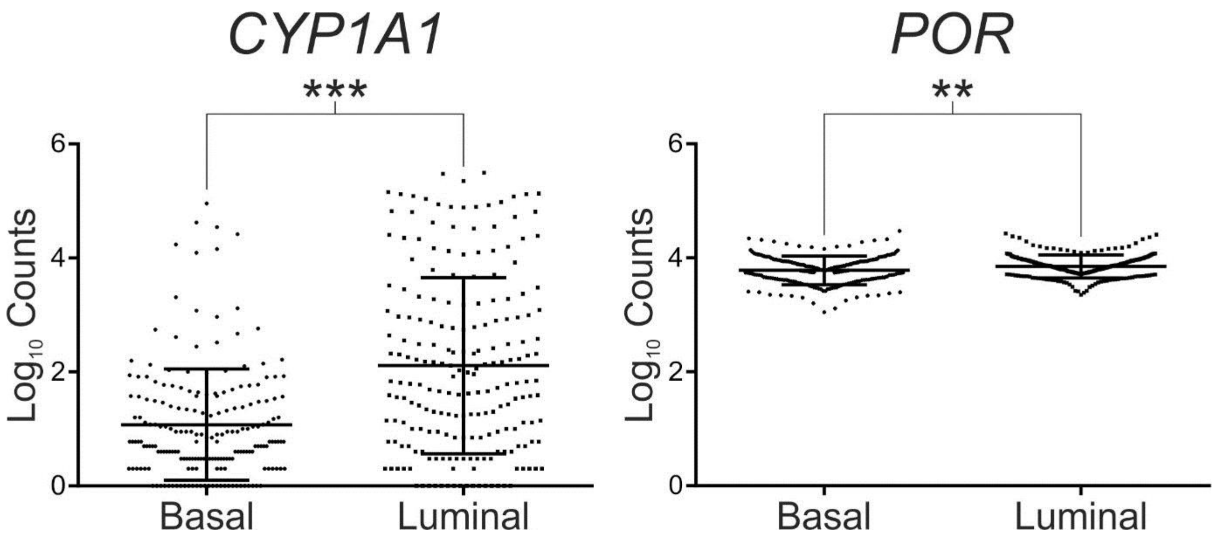

Supplementary Figure 9 – *CYP1A1* and *POR* transcript expression quantified from RNA sequencing data of the The Cancer Genome Atlas consortium and separated into basal and luminal subtypes based on the gene classifier reported by Choi *et al.* (55). Both CYP1A1 and POR transcripts were significantly elevated in the luminal group of tumours as assessed by Mann Whitney U test.

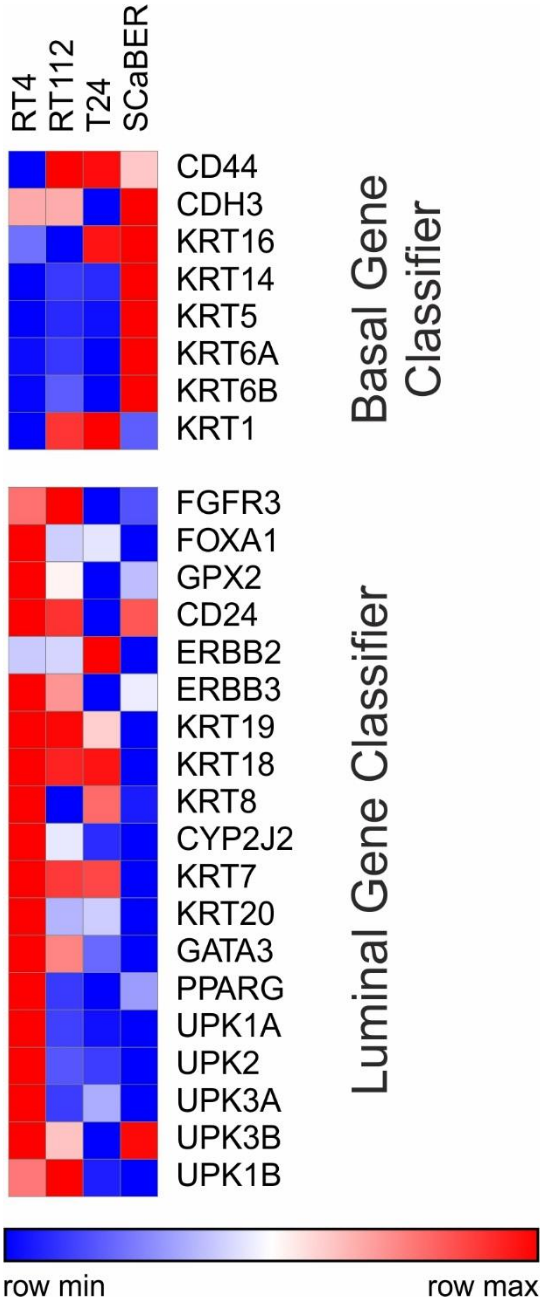

Supplementary Figure 10 – Heatmap of "UBC-40" bladder cancer cell line gene array data (54) for the relative expression of Choi *et al.* (55) gene classifiers by RT4, RT112, T24 and SCaBER cells. RT4 and RT112 were more differentiated ("luminal"). T24 and SCaBER were more "basal" or undifferentiated.

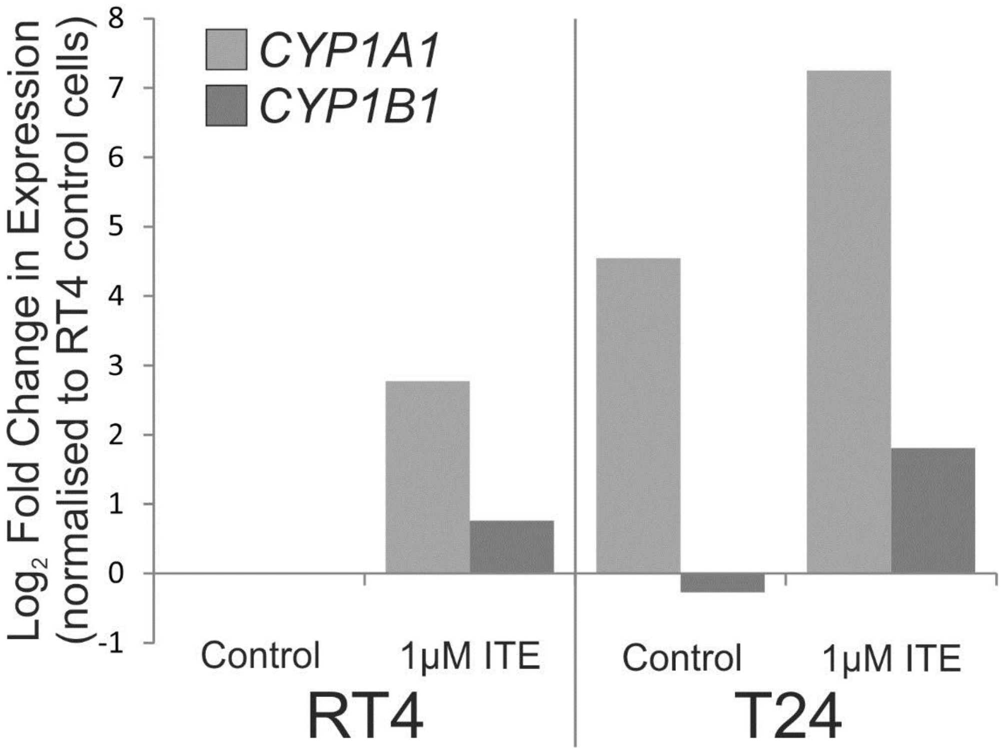

Supplementary Figure 11 – RT-qPCR demonstrated that 24 h 1 µM ITE exposure induced *CYP1A1* and *CYP1B1* transcript in both RT4 and T24 cells. Both baseline and induced expression of *CYP1A1* transcript was higher in T24 cells (*n* = 1).

```
Mouse        ITNGKSMTFNPDSGPVWAARRRLAQNALKSFSIASDPTSASSCYLEEHVSKEANYLVSKL
Rat          IANGQSMTFNPDSGPLWAARRRLAQNALKSFSIASDPTLASSCYLEEHVSKEAEYLISKF
Human        ISNGQSMSFSPDSGPVWAARRRLAQNGLKSFSIASDPASSTSCYLEEHVSKEAEVLISTL
Pig          ISNGQSMTFNPDSGPVWAARRRLAQKALNTFSIASDPASSSSCYLEDHVSKEAECLLGKF
             *:**:**:*.*****:*********:.*::*******: ::*****:******: *:..:

Mouse        QKVMAEVGHFDPYKYLVVSVANVICAICFGQRYDHDDQELLSIVNLSNEFGEVTGSGYPA
Rat          QKLMAEVGHFDPFKYLVVSVANVICAICFGRRYDHDDQELLSIVNLSNEFGEVTGSGYPA
Human        QELMAGPGHFNPYRYVVVSVTNVICAICFGRRYDHNHQELLSLVNLNNNFGEVVGSGNPA
Pig          QELMAGPGHFDPYKYVLVSVANVICAICFGQRYDHDNPELLSLINLSNEFGEVTAAGNPA
             *::**  ***:*::*::***:*********:****:. ****::**.*:****..:* **

Mouse        DFIPVLRYLPNSSLDAFKDLNDKFYSFMKKLIKEHYRTFEKGHIRDITDSLIEHCQDRKL
Rat          DFIPILRYLPNSSLDAFKDLNKKFYSFMKKLIKEHYRTFEKGHIRDITDSLIEHCQDRRL
Human        DFIPILRYLPNPSLNAFKDLNEKFYSFMQKMVKEHYKTFEKGHIRDITDSLIEHCQEKQL
Pig          DFIPILRYLPNTSLDLFKDLNQKFYIFMQKMVREHYKIFEKGRIRDITDSLIEHCQDKRM
             ****:****** **: *****.*** **:*:::***: ****:*************::::

Mouse        DENANVQLSDDKVITIVLDLFGAGFDTVTTAISWSLMYLVTNPRVQRKIQEELDTVIGRD
Rat          DENANVQLSDDKVITIVFDLFGAGFDTITTAISWSLMYLVTNPRIQRKIQEELDTVIGRD
Human        DENANVQLSDEKIINIVLDLFGAGFDTVTTAISWSLMYLVMNPRVQRKIQEELDTVIGRS
Pig          DENANIQLSDEKIVNIVIDLFGAGFDTVTTAISWSLMYLVTNPSIQRKIQEELDTVIGQA
             *****:****:*::.**:*********:************ ** :*************:

Mouse        RQPRLSDRPQLPYLEAFILETFRHSSFVPFTIPHSTTRDTSLNGFYIPKGCCVFVNQWQV
Rat          RQPRLSDRPQLPYLEAFILETFRHSSFVPFTIPHSTIRDTSLNGFYIPKGHCVFVNQWQV
Human        RRPRLSDRSHLPYMEAFILETFRHSSFVPFTIPHSTTRDTSLKGFYIPKGRCVFVNQWQI
Pig          RRPRLSDRPQLPYMEAFILELFRHTSFVPFTIPHSTTRDTSLNGFYIPKGRCVFVNQWQI
             *:****** :***:****** ***:*********** *****:******* ********:

Mouse        NHDRELWGDPNEFRPERFLTPSGTLDKRLSEKVTLFGLGKRKCIGETIGRSEVFLFLAIL
Rat          NHDQELWGDPNEFRPERFLTSSGTLDKHLSEKVILFGLGKRKCIGETIGRLEVFLFLAIL
Human        NHDQKLWVNPSEFLPERFLTPDGAIDKVLSEKVIIFGMGKRKCIGETIARWEVFLFLAIL
Pig          NHDQKLWDDPSVFRPERFLTADGTINKALGEKVILFGLGKRKCIGETIARLEVFLFLAIL
             ***::** :*. * ****** .*:::* *.*** :**:**********.* *********

Mouse        LQQIEFKVSPGEKVDMTPTYGL
Rat          LQQMEFNVSPGEKVDMTPAYGL
Human        LQRVEFSVPLGVKVDMTPIYGL
Pig          LQQVEFRVTPGVKVDMTPIYGL
             **::** *  * ****** ***
```

1: Mouse	100.00	93.19	80.89	80.10
2: Rat	93.19	100.00	80.63	81.15
3: Human	80.89	80.63	100.00	82.46
4: Pig	80.10	81.15	82.46	100.00

Supplementary Figure 12 – Clustal Omega alignment of Ensembl protein sequences for the BaP interacting region of CYP1A1 in human (amino acids 115-496 [49]; ENST00000379727.7), CL57BL6 mouse (ENSMUST00000216433.1), rat (ENSRNOT00000026473.4) and pig (ENSSSCT00000002135.3). The alignment highlights, in bold and underlined text, the (uncharged) asparagine at amino acid 222 in the human protein which is modified to an aspartic acid (negatively charged) in the experimental animals.

Supplementary Methods

Western Blotting

Whole cell lysates were obtained from cultures by *in situ* lysis in sodium dodecyl sulphate (SDS) electrophoresis sample buffer, containing 13 mM dithiothreitol (DTT) and 0.2% (*v*/*v*) protease inhibitors (Protease Inhibitor Cocktail set III, Calbiochem). Lysates were sonicated, microcentrifuged and the protein concentration determined by Bradford colorimetric assay.

For all proteins except CYP1A1 and GAPDH the method was as follows. Protein samples (20 µg) were resolved by electrophoresis of NuPAGE gels using the Novex system (Invitrogen) at 200 V. Total protein separated by sodium dodecyl sulfate polyacrylamide gel electrophoresis (SDS-PAGE) was electro-transferred to PVDF-FL membranes (Milipore) in a Tris–glycine buffer at 30 V for 3 h at 4°C. Membranes were blocked for 1 h in Odyssey blocking buffer (LI-COR), incubated with titrated primary antibody overnight at 4°C. A rabbit anti-Ahr/AHR affinity-purified polyclonal was used at a dilution of 1:2,000 (Enzo Life Sciences, BML-SA210). NADPH:P450 oxidoreductase (POR) rabbit polyclonal "CH60" antibody was a kind gift from Prof Roland Wolf and Dr Colin Henderson (Dundee University) and used at a dilution of 1:10,000 [48]. Homogeneous loading and transfer was evaluated using β-actin (Sigma, Clone AC15, Mouse, 1:10,000 dilution) intensity. Membranes were labelled with the appropriate IRDye conjugated secondary antibody (LI-COR) at ambient temperature for 1 h and visualised by epifluorescent infrared illumination at 700 and/or 800 nm using the Odyssey scanner and software (LI-COR).

Detection of human CYP1A1 and GAPDH protein was performed as described [49] and not according to the method above. CYP1A1 antibody raised in rabbits against purified human recombinant CYP1A1 was a generous gift from Prof F. Peter Guengerich (Vanderbilt University, USA) and used at a dilution of 1:4000.

All Densitometry was performed using Image Studio Lite version 5.0 (LI-COR Biosciences).

Reverse Transcribed - quantitative Polymerase Chain Reaction (RT-qPCR)

The relative abundance of selected transcripts was assessed using the following forward and reverse primers (all 5'-3') to amplify *CYP1A1* (ACCAAGAACTGCTTAGCCTAGTCAA and GGTTGGGTAGGTAGCGAAGAATAG), *CYP1B1* (CCAGCTTTGTGCCTGTCACTAT and GGGAATGTGGTAGCCCAAGA), *POR* (AGCATGACGGACATGATTCTGT and TCAATGTCTGAATTTTGGTGAACTC) and *GAPDH* (CAAGGTCATCCATGACAACTTTG and GGGCCATCCACAGTCTTCTG). Primers were optimised to give a linear response over a 1,000 fold dilution range and a single product as characterised by a single peak in the dissociation curve. Amplification was monitored using SYBR Green dye on an ABI7300 machine (Applied Biosystems). All measurements were performed in triplicate and calculated using the ΔΔct method relative to *GAPDH* expression. Total human liver mRNA from pooled donors (Agilent) was used as a reference to normalise gene expression.

Immunoperoxidase labelling

Bladders from *Ahr*-knockout and wild-type C57BL/6 male mice aged 6-12 weeks were a kind gift from Prof Roland Wolf [50]. Tissues were fixed in 10% formalin and processed into paraffin wax. Sections (5 µm) were collected onto Superfrost™ Plus slides (VWR), dewaxed in xylene and rehydrated through ethanol to water. Antigen retrieval was performed by boiling slides in 10 mM citric acid buffer (pH 6) for 10 min (for Ahr/AHR labelling 0.1% (*w*/*v*) NP40 was added to the buffer). Endogenous avidin and biotin binding sites were blocked (Vector) and non-specific antibody binding was prevented by 10% (*v*/*v*) rabbit or goat serum (Dako), depending on the host of the secondary antibody. Tissues were incubated with primary antibodies raised against AhR (details as per western blotting, 1:2,000 dilution), Claudin 5 (Invitrogen, Clone 4C3C2, Mouse, 10 µg/mL) and Uroplakin 3a (Progen, Clone AU1, Mouse, 1:40 dilution) at 4°C overnight. Appropriate biotinylated secondary anti-immunoglobulin antibody (Dako) was applied for 30 min at ambient temperature, then streptavidin–biotin–horseradish peroxidase complex (Vector), with washing between each step. Bound antibody was detected using diaminobenzidine (Sigma) and slides were counterstained with Mayer's haematoxylin before mounting in DPX.

Trans-Epithelial Electrical Resistance (TEER) Monitoring Method

Following preconditioning for 5 days in 5% adult bovine serum (ABS) cells were harvested and reseeded onto 1.13 cm^2 permeable Snapwell^{TM} supports (Corning) at $5x10^5$ cells per membrane. After 24 h, the exogeneous calcium concentration was increased to 2 mM and the cultures maintained for 7 days before treatment and TEER was monitored using a portable Epithelial Volt-ohmmeter (World Precision Instruments). 1 µM ITE treatment was applied throughout the differentiation process, including preconditioning.

Analysis of The Cancer Genome Atlas (TCGA) cohort of MIBC

RNA sequencing data for TCGA cohort of MIBC [51] was downloaded from the NIH Genome Data Commons as normalised counts data and log transformed. MIBC was classified as either luminal or basal as previously described [52].

Basal/Luminal Classification of Cancer Cell Line Models

From previously published gene array data for a collection of bladder cancer cell lines [53], the data for RT4, RT112, T24 and SCaBER cells was extracted. The Choi *et al.* molecular classification of luminal and basal muscle invasive bladder cancers describes two gene set “classifiers” [52] and those genes were extracted and displayed as a heatmap.

Supplementary References

48. Smith GC, Tew DG, Wolf CR. Dissection of NADPH-cytochrome P450 oxidoreductase into distinct functional domains. PNAS 1994;91(18):8710-8714.
49. Wohak LE, Krais AM, Kucab JE et al. Carcinogenic polycyclic aromatic hydrocarbons induce CYP1A1 in human cells via a p53-dependent mechanism. Archives of toxicology 2016;90(2):291-304.
50. Fernandez-Salguero P, Pineau T, Hilbert DM et al. Immune system impairment and hepatic fibrosis in mice lacking the dioxin-binding Ah receptor. Science 1995;268(5211):722-726.
51. TCGARN. Comprehensive molecular characterization of urothelial bladder carcinoma. Nature 2014;507(7492):315-322.

52. Choi W, Porten S, Kim S et al. Identification of distinct basal and luminal subtypes of muscle-invasive bladder cancer with different sensitivities to frontline chemotherapy. Cancer Cell 2014;25(2):152-165.

53. Earl J, Rico D, Carrillo-de-Santa-Pau E et al. The UBC-40 Urothelial Bladder Cancer cell line index: a genomic resource for functional studies. BMC Genomics 2015;16:403.
