# Supplementary material for: Differentiation‐associated urothelial cytochrome P450 oxidoreductase predicates the xenobiotic‐metabolizing activity of “luminal” muscle‐invasive bladder cancers
Source: Mol Carcinog. 2018 Feb 1;57(5):606–18. doi: 10.1002/mc.22784 (PMC5900743; doi:10.1002/mc.22784)
Supplement: Supplementary file 2 — Table S1. Clinical characteristics and histological assessment of MIBC specimens included in the study. [file MC-57-606-s002.pdf]

Supplementary Table 1 – Clinical characteristics and histological assessment of MIBC specimens included in the study.

POR Labelling Intensity	Classified as POR over-expressing	GATA3 Postive Nuclei (%)	KRT5/6-positive tissue (%)	Classified as Basal or Luminal	Year of cystectomy	Age at cystectomy	Gender	2010 TNM Tumour Stage	1973 WHO Grading	Number of Lymph Nodes Involved	Clinical Signs of Metastasis
147.00	Yes	47.58	Sample Damaged	Not classified (no KRT5/6)	2001	75	Male	6	3	2	Yes
122.67	Yes	84.90	15.52	Luminal	2005	75	Male	6	3	2	No
106.00	Yes	86.25	97.68	Luminal	2005	71	Male	5	3	0	No
101.00	Yes	79.48	0.20	Luminal	2002	69	Male	5	3	2	No
98.00	Yes	49.96	50.47	Luminal	2004	71	Male	6	3	1	Yes
81.00	Yes	95.02	0.24	Luminal	2004	61	Male	6	3	2	No
79.00	Yes	90.79	88.98	Luminal	2005	72	Female	5	3	0	No
72.00	Yes	79.94	28.35	Luminal	2001	60	Male	5	3	2	No
68.00	Yes	91.29	0.00	Luminal	2006	53	Male	6	3	2	No
58.25	No	87.93	44.37	Luminal	2006	78	Male	6	3	2	No
57.25	No	34.86	71.89	Basal	2000	83	Male	6	3	2	No
53.00	No	98.49	0.00	Luminal	2005	62	Female	4	3	0	No
52.00	No	93.42	4.21	Luminal	2006	75	Male	6	3	2	Yes
51.00	No	39.23	0.93	Luminal	2000	76	Male	6	3	2	No
46.00	No	94.12	0.00	Luminal	2003	67	Male	6	3	2	No
45.00	No	89.02	0.00	Luminal	2005	73	Female	5	3	0	No
43.00	No	12.45	0.00	Luminal	2007	74	Male	5	3	1	Yes
42.50	No	87.43	0.00	Luminal	2005	55	Male	5	3	0	No
41.00	No	79.26	0.00	Luminal	1998	81	Male	5	3	1	Yes
38.00	No	70.39	23.76	Luminal	2000	72	Male	3	3	1	No
36.50	No	48.54	85.13	Basal	2005	68	Male	5	3	0	No
36.00	No	22.35	91.32	Basal	2003	50	Female	5	3	2	No
36.00	No	2.35	0.00	Luminal	2007	78	Male	6	3	0	No
34.33	No	95.90	0.11	Luminal	2004	80	Male	0		2	No
32.00	No	50.12	0.00	Luminal	1999	59	Male	5	3	0	No
31.20	No	85.63	2.49	Luminal	2008	83	Male	6	3		
31.00	No	77.84	0.04	Luminal	2001	79	Male	6	3	2	No

26.50	No	87.55	5.23	Luminal	2003	76	Male	5	3	2	No
25.00	No	75.15	3.23	Luminal	2003	56	Male	6	3	1	Yes
24.00	No	7.56	0.01	Luminal	2002	73	Male	4	3	1	No
24.00	No	89.65	0.00	Luminal	2005	55	Female	5	3	1	No
23.00	No	94.11	0.00	Luminal	2006	81	Male	5	3	1	No
22.00	No	94.03	0.00	Luminal	2006	66	Male	5	3	2	No
22.00	No	58.51	0.00	Luminal	2008	80	Female	6	3	1	No
22.00	No	39.91	35.77	Luminal	2007	83	Female	5	3	0	No
21.86	No	0.68	55.49	Basal	1996	83	Male	5	3		
21.50	No	83.72	4.81	Luminal	2000	48	Male	6	3	2	No
21.25	No	94.74	0.03	Luminal	2002	79	Female	6	3	0	Yes
21.00	No	90.59	0.00	Luminal	2003	56	Male	6	3	1	Yes
20.17	No	32.65	0.32	Luminal	2006	73	Female	5	3	2	No
18.50	No	72.57	0.62	Luminal	2004	97	Male	6	3	1	Yes
18.50	No	1.36	2.65	Luminal	1998	60	Male	5	3	2	No
17.33	No	98.16	0.11	Luminal	2005	81	Male	6	3	2	No
17.00	No	96.42	0.00	Luminal	2003	67	Male	6	3	2	No
16.75	No	0.99	47.44	Basal	2008	66	Male	5	3	2	No
16.00	No	87.89	0.00	Luminal	2004	70	Male	5	3	2	No
15.13	No	74.10	0.21	Luminal	2005	81	Male	5	3	2	No
14.80	No	33.24	0.00	Luminal	2007	70	Male	6	3	2	Yes
14.67	No	1.14	52.51	Basal	1998	67	Male	6	3	2	No
14.50	No	10.65	18.68	Luminal	2007	70	Male	5	3	1	No
14.00	No	1.34	47.73	Basal	1999	65	Male	6	3	2	Yes
13.50	No	90.16	1.50	Luminal	2007	67	Male	6	3	1	No
13.50	No	14.27	59.45	Basal	2004	80	Female	5	3	2	No
12.50	No	0.05	72.21	Basal	2006	71	Female	6	3	2	No
12.50	No	67.36	0.40	Luminal	2008	74	Male	5	3	1	No
12.25	No	85.86	0.00	Luminal	2000	74	Male	6	3	0	Yes
11.67	No	7.74	33.79	Basal	2007	61	Male	5	3	1	No
10.40	No	72.28	1.86	Luminal	2008	72	Male	5	3	2	No
10.00	No	1.43	98.34	Basal	2003	64	Female	6	3	1	No
0.00	No	0.00	0.01	Luminal	1998	59	Male	5	3	2	No
